# Discovery of DNA dyes Hoechst 34580 and 33342 as good candidates for inhibiting amyloid beta formation: in silico and in vitro study

**DOI:** 10.1007/s10822-016-9932-1

**Published:** 2016-08-10

**Authors:** Nguyen Quoc Thai, Ning-Hsuan Tseng, Mui Thi Vu, Tin Trung Nguyen, Huynh Quang Linh, Chin-Kun Hu, Yun-Ru Chen, Mai Suan Li

**Affiliations:** 1 Institute for Computational Science and Technology, SBI Building, Quang Trung Software City, Tan Chanh Hiep Ward, District 12, Ho Chi Minh City, Vietnam; 2 Biomedical Engineering Department, University of Technology -VNU HCM, 268 Ly Thuong Kiet Str., Distr. 10, Ho Chi Minh City, Vietnam; 3 Division of Theoretical Physics, Dong Thap University, 783 Pham Huu Lau Street, Ward 6, Cao Lanh City, Dong Thap Vietnam; 4 Genomics Research Center, Academia Sinica, Academia Rd., Sec. 2, Nankang Dist., Taipei 115, Taiwan; 5 Institute of Physics, Academia Sinica, 128 Academia Road Section 2, Taipei, 11529 Taiwan; 6 National Center for Theoretical Sciences, National Tsing Hua University, 101 Kuang-Fu Road Section 2, Hsinch, 30013 Taiwan; 7 Business School, University of Shanghai for Science and Technology, 334 Jun Gong Road, Shanghai, 200093 China; 8 Institute of Physics, Polish Academy of Sciences, Al. Lotnikow 32/46, 02-668 Warsaw, Poland

**Keywords:** Alzheimer’s disease, DNA dyes, Hoechst 34580, Hoechst 33342, Drug design, Amyloid beta fibril

## Abstract

Combining Lipinski’s rule with the docking and steered molecular dynamics simulations and using the PubChem data base of about 1.4 million compounds, we have obtained DNA dyes Hoechst 34580 and Hoechst 33342 as top-leads for the Alzheimer’s disease. The binding properties of these ligands to amyloid beta (Aβ) fibril were thoroughly studied by in silico and in vitro experiments. Hoechst 34580 and Hoechst 33342 prefer to locate near hydrophobic regions with binding affinity mainly governed by the van der Waals interaction. By the Thioflavin T assay, it was found that the inhibition constant IC50 ≈ 0.86 and 0.68 μM for Hoechst 34580 and Hoechst 33342, respectively. This result qualitatively agrees with the binding free energy estimated using the molecular mechanic-Poisson Boltzmann surface area method and all-atom simulations with the AMBER-f99SB-ILDN force field and water model TIP3P. In addition, DNA dyes have the high capability to cross the blood brain barrier. Thus, both in silico and in vitro experiments have shown that Hoechst 34580 and 33342 are good candidates for treating the Alzheimer’s disease by inhibiting Aβ formation.

**Electronic supplementary material:**

The online version of this article (doi:10.1007/s10822-016-9932-1) contains supplementary material, which is available to authorized users.

## Introduction

Alzheimer’s disease (AD) is one of the most common forms of dementia [1]. Clinically it is defined as a progressive decline in memory, language and other cognitive functions. AD is the sixth-leading cause of death in the United States and total payments for patients with AD and other dementias are estimated at $226 billion in 2015 [2] posing huge burden to the society. Despite intense research during many decades, the problem of finding efficient drugs for AD remains challenging. Available drugs which are acetylcholinesterase inhibitors and N-methyl-D-aspartate (NMDA) receptor antagonists can treat some symptoms but not cure the disease.

In order to design potential drugs for a given disease one has to know the corresponding target but such a target for AD remains largely uncertain as the cause of AD has not been disclosed yet [3]. There are about twenty hypotheses concerning AD mechanisms [4], but recent experimental evidences strongly support the amyloid cascade hypothesis [5] positing that AD is associated with progressive intra-cerebral accumulation of beta amyloid (Aβ) peptides [6]. In addition, oligomers are presumably more toxic that mature fibrils [7, 8]. Because Aβ peptides are generated by the proteolytic cleavage of amyloid precursor protein (APP) by β- and γ-secretases, AD can be cured by either blocking activity of these secretases or preventing Aβ aggregation. In the latter case Aβ oligomers or fibrils become the drug target. Following this strategy a lot of potential Aβ inhibitors have been identified including short peptides [9], nutraceuticals [10–13], polyamines [14, 15], metal chelators [16], derivatives of vitamin K3 [17], RNA aptamers [18], osmolytes [19], and other compounds [15, 20, 21].

In the present paper, we have carried out the multi-step screening of Aβ aggregation inhibitors from data basic PubChem [22] (http://pubchem.ncbi.nlm.nih.gov) using the Lipinski’s rule [23, 24] in combination with the molecular docking and steered molecular dynamic (SMD) simulations. From predicted top-leads for Aβ_40_ and Aβ_42_ fibrils we succeeded to purchase DNA dyes Hoechst 34580 and Hoechst 33342 for in vitro experiment. Using the Thioflavin T (ThT) assay the inhibition constant IC50 was found to be equal 0.86 and 0.68 μM for Hoechst 34580 and Hoechst 33342, respectively. This result is consistent with our estimation of the absolute binding free energy by the molecular mechanic Poisson–Boltzmann surface area (MM-PBSA) [25] method which is more accurate than the docking method. In addition, the QSAR analysis revealed that both DNA dyes are capable to easily cross the blood brain barrier (BBB) implying that they are good candidates for AD treatment.

## Materials and methods

### Data base of ligands and receptors

Screening of drug candidates has been performed using about 1.4 million compounds from Collaborative Drug Discovery in PubChem [22] (see http://pubchem.ncbi.nlm.nih.gov). Concerning the target (receptor) we chose the structural model of Aβ40 and Aβ42 fibrils. For Aβ_40_ fibril, the model of truncated fragment Aβ_9-40_ which is available in the Protein Data Bank with PDB ID: 2LMN [26] with the 8 first disordered residues neglected. This structure has two layers each of which contains 6 strands numbered as A-F and G-L (Figure S1 in Supporting Information (SI)). A full molecular structural model for Aβ40 fibril is the three-fold-symmetric (PDB ID: 2M4J [27]) containing 9 chains (Figure S1). For Aβ42 fibril, the structure of truncated fragment Aβ17-42 (PDB ID: 2BEG_ENREF_25 [28]), which is a model built basically on mutagenesis and H/D exchange experiments, and the solid-state NMR structures of Aβ11-42 (PDB ID 2MXU [29]) were employed (Figure S1).

### Lipinski’s rule

**Fig. 1:**
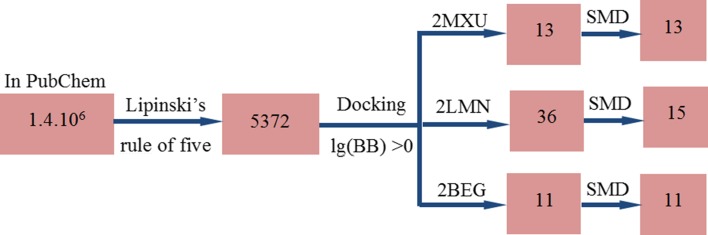
Multi-step screening procedure. From 1.4 million compounds we keep only 5372 compounds satisfying the Lipinski’s rule for drug-like ligands. The further screening by docking method and requirement that drug candidates should have the binding energy ΔEbind < −9.0 kcal/mol and lg(BB) > 0 give the set of 27 ligands for 2MXU, and binding energy ΔEbind < −10.0 kcal/mol and lg(BB) > 0 give the set of 36 ligands for 2LMN. Imposing that candidates should have ΔEbind < −8 kcal/mol and lg(BB) > 0 we obtained 11 ligands for 2BEG, respectively. Applying the SMD method to the set of 36 ligands we obtained 15 top leads for 2LMN and 13 top leads for 2MXU, while this method was just used for re-ranking 11 top leads for 2BEG

First, ligands were virtually screened by Lipinski’s rule of five [23]. It sets for drug-like properties [23, 24] as molecular weight from 0 to 500 Da, xlogP from 0 to 5, the number of donor hydrogen bonds is from 0 to 5, and the number of acceptor hydrogen bonds is from 0 to 10. The application of Lipinski’s rule reduced the whole set of about 1.4 million compounds to 5372 compounds (Fig. 1).

### Docking method

Autodock Tool 1.5.4 [30] was used to prepare PDBQT file for docking ligands to targets 2LMN, 2BEG, 2MXU, and 2M4J. The docking simulation was performed using the Autodock Vina version 1.1 [31]. For global search, the exhaustiveness was set to 400 which is enough for obtaining reasonable results. Twenty binding modes have been generated starting from random configurations of ligand which had fully flexible torsion degrees of freedom. Because the binding site of Aβ fibrils was not a priori known to cover the whole fibril the boxes of grid dimensions 4.7 × 5.3 × 7.4 nm (2LMN), 2.9 × 4.6 × 25 nm (2BEG), 5.3 × 4.2 × 6.5 nm (2MXU) and 9.2 × 9.2 × 3.5 nm (2M4J) were chosen. The dynamics of receptor was neglected. The lowest binding energy Δ*E*_bind_ obtained in the best docking mode was chosen as a scoring function for selecting top ligands. Here we selected only those ligands which have Δ*E*_bind_ less than −10.0, −8.0 and −9.0 kcal/mol for targets 2LMN, 2BEG, and 2MXU, respectively.

### Molecular dynamic (MD) simulation

To estimate the binding free energy by the MM-PBSA method, the molecular dynamics simulation was carried out with the AMBER-f99SB-ILDN force field [32] and water model TIP3P [33]. The rationale for our choice of AMBER-f99SB-ILDN is that this force field, as shown by previous work [9, 34–36], provided reasonable results on binding affinity of small molecules to amyloid fibrils. The GAFF force field [37] was used for parameterization of ligands Hoechst 33342 and Hoechst 34580. Restrained electrostatic potential [38] (RESP) point charges were assigned to ligand atoms by the Antechamber package [39] based on electrostatic potential (ESP) calculated by Gaussian09 package [40] at the B3LYP/6-31G* level. Names, types atoms, masses and charges of atoms used in the simulation for Hoechst 33342 and Hoechst 34580 are listed in Table S1 and S2 in SI.

The fibril-ligand complex was placed in the 9.3 × 9.3 × 9.3 (2LMN), 6.8 × 6.8 × 6.8 (2BEG), 7.6 × 7.6 × 7.6 (2MXU) and 11.1 × 11.1 × 11.1 nm^3^ (2M4J) cubic boxes containing about 78,500, 31,400, 41,448 and 136,200 water molecules with 1 nm distance between the box and solute. The van der Waals (vdW) forces were calculated with a cutoff of 1.4 nm, while the long-range electrostatic interaction was computed by the particle-mesh Ewald summation method [41]. Equations of motion were iterated by a leapfrog algorithm [42] with a time step 2 fs. The overall charge of the systems was set to zero by adding 12, 5, 8 and 27 Na^+^ ions to 2LMN, 2BEG, 2MXU and 2M4J, respectively. After minimization by the steepest descent method, the position-restrained MD simulations were performed for 500 ps to let water molecules to move into the active site. The equilibration was reached by coupling with temperature and pressure. Constant temperature 300 K was kept using Langevin dynamics with the collision frequency of 2.0 ps^−1^. The Berendsen barostat [43] was used to maintain the pressure at 1 atm and 300 K with the pressure relaxation time of 1.0 ps.

### Steered molecular dynamics

The steered molecular dynamics (SMD) method was developed to study mechanical unfolding of biomolecules [44, 45] and ligand unbinding from receptor along a given direction [46]. Recently, it has been shown that this method is as accurate as the MM-PBSA method but computationally much less demanding [47, 48]. Because the predictive power of the docking method is limited the SMD method was employed to refine docking results as a next step in the multi-step screening procedure. Overall, an spring with spring constant k is attached to a dummy atom at one end and to the first heavy atom of ligand in the pulling direction at the another end. Moving along the pulling direction with a constant loading rate *v* the dummy atom experiences elastic force *F* = *k*(∆*x* − *vt*), where ∆*x* is the displacement of pulled atom from the starting position. We have chosen the spring constant *k* = 600 kJ/(mol nm^2^) which is a typical value for cantilever used in AFM experiment [49]. As in our previous works [48, 50, 51], the loading speed was set equal *v* = 5 nm/ns. This choice of parameters *k* and *v* was proved as reasonable for pulling experiment [51]. All Cα-atoms of receptor were restrained to keep the receptor almost at the same place but still maximally maintain its flexibility.

We determined possible pathways of ligands by using CAVER 3.0 [52], Pymol plugin, and chose the easiest path for ligand to exit from receptor as the pulling direction [50]. After equilibration, to completely pull the ligand out of the binding site, 500 ps SMD runs were carried out in NPT ensemble. To obtain reliable results five independent trajectories were performed with different random seeds. In the SMD method the maximum force *F*_max_ in the force-extension/time profile was chosen as a score for binding affinity, i.e. the larger is *F*_max,_ the stronger is the ligand binding.

### MM-PBSA method

The MM-PBSA method [25] was used to estimate the binding free energy Δ*G*_bind_ of DNA dyes to targets 12Aβ9-40 (2LMN), 9Aβ1-40 (2M4J), 5Aβ17-42 (2BEG), and 8Aβ11-42 (2MXU). More details on this method may be found elsewhere [53, 54]. Typically, Δ*G*_bind_ is given by the following expression:

1 $$\Delta G_{\text{bind}} = \Delta E_{\text{elec}} + \Delta E_{\text{vdW}} + \Delta G_{\text{sur}} + \Delta G_{\text{PB}} - T\Delta S,$$

where ∆*E*_elec_ and ∆*E*_vdW_ are contributions from electrostatic and vdW interactions, respectively. ∆*G*_sur_ and ∆*G*_PB_ are nonpolar and polar solvation energies. The entropic contribution *T*∆*S* was estimated using the normal mode approximation. Snapshots collected in equilibrium and Eq. (1) were used to compute Δ*G*_bind_.

### Blood brain barrier

One of the most important requirements for AD drug candidates is that they should be able to cross the blood brain barrier (BBB) [55] which is created by the brain capillary endothelium. The logarithm base 10 of the ratio of the ligands concentration in the brain to that in the blood, log(BB), is a measure of capability of a given ligand to pass BBB. This quantity is estimated by the QSAR (quantitative structure–activity relationship) approach [56]. The sever preADMET [57, 58] (see http://preadmet.bmdrc.org/) was used to calculate log (BB).

### Measures used in data analysis

The backbone root mean square deviation (RMSD), computed using the Gromacs 5.1 package, was used to measure the deviation of structures of the receptor from its initial configuration. A hydrogen bond (HB) was formed provided the distance between donor D and acceptor A is less than 3.5 Å, the H-A distance is less than 2.7 Å and the D-H-A angle is larger than 135 degrees.

### Aβ preparation

Aβ was prepared as described in previous literatures [10]. Briefly, Aβ42 peptide (Biopeptide, San Diego, CA) was dissolved in 50 % acetonitrile, divided into aliquots, lyophilized overnight, and stored at −80 °C. Before experiments, Aβ42 was dissolved in hexafluoroisopropanol (HFIP) in 1 mg/mL. The sample was mixed vigorously using a vortex for 5 s and then sonicated for 5 min. After quiescent for 1 h, the HFIP was evaporated in vacuum and Aβ peptides were dissolved by anhydrous dimethyl sulfoxide (DMSO) in 60 mg/mL and then diluted in 10 mM phosphate buffer, pH 7.4. The final Aβ concentration was 50 μM in ThT assays and 25 μM in fluorescence titration.

### Compounds preparation

As seen below, Hoechst 34580 and Hoechst 33342 are among the top leads revealed by molecular simulation. They were purchased from Sigma-Aldrich (St. Louis, MD) and were used without purification. Compounds were dissolved respectively in DMSO at 10 mM as stocks. In ThT assays, the desired concentrations of the compounds were serial diluted in DMSO. In fluorescence titration assay, 10 mM compounds were diluted with 10 mM phosphate buffer (pH 7.4) to 5 mM and proceed for titration.

### Structures of Hoechst 34580 and Hoechst 33342

**Fig. 2:**
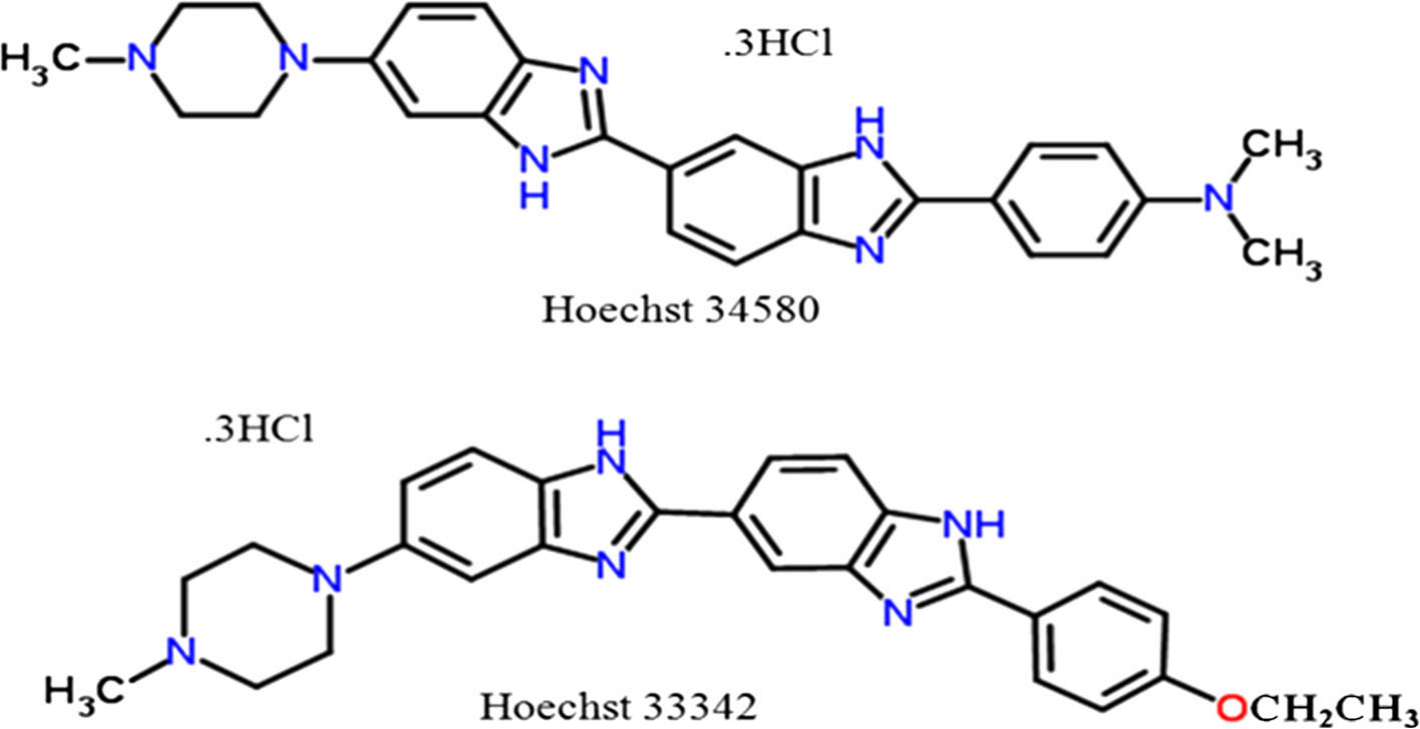
2D structures of Hoechst 34580 and Hoechst 33342

In this study, Hoechst 34580 and Hoechst 33342 which are commercially available cell-permeable fluorescent dye for staining DNA and nuclei (Fig. 2). They are identical except the last fragment NCH_3_CH_3_ for Hoechst 34580 and OCH_2_CH_3_ for Hoechst 33342.

### ThT assay

Aβ42 fibrillization was measured using a Thioflavin T (ThT) assay. The compounds, from 1.22 μM to 10 mM, were prepared in DMSO and 0.4 μl of each was added to 384 well black plate. Each concentration was prepared in independent triplicates and a solvent control was included. Aβ42 solution at 50 μM in 39.6 μl was prepared with addition of 5 μM ThT. The samples were incubated at 37 °C with agitation for 1 min every hour. ThT fluorescence was monitored using an ELISA microplate reader SpectraMax M5 (Molecular Devices, Sunnyvale, CA) at an excitation wavelength of 442 nm and an emission wavelength of 485 nm. Measurements from independent triplicate trials were averaged and the standard deviations were calculated.

## Results and discussion

### Simulation results

#### Docking results

After the first virtual screening step by Lipinski’s rule, the number of compounds is reduced to 5372 (Fig. 1). The Autodock Vina [31] method was then applied to dock this set to targets 2LMN, 2BEG, and 2MXU. We did not perform the similar docking simulation for the whole ligand set to 2M4J because both 2M4J and 2MXU are solid state NMR structures. However, the docking of DNA dyes was carried for the target 2M4J as well. The binding energies Δ*E*_bind_, obtained in the best docking modes for 5327 ligands, vary from −0.6 to −11.4 (2LMN), −1.2 to −8.8 (2BEG), and −1.4 to −11.9 kcal/mol (2MXU) (Figure S2 in SI). There are 96 compounds that have the binding energy lower than −10 kcal/mol for 2LMN, 55 **c**ompounds have Δ*E*_bind_ < **−**8.0 kcal/mol for 2BEG and 57 compounds have Δ*E*_bind_ < −9.0 kcal/mol for 2MXU. Locations of these compounds in fibrils are presented in Figure S3 in SI. As expected Aβ do not have well defined binding sites because ligands locate either inside or between two layers.

**Fig. 3:**
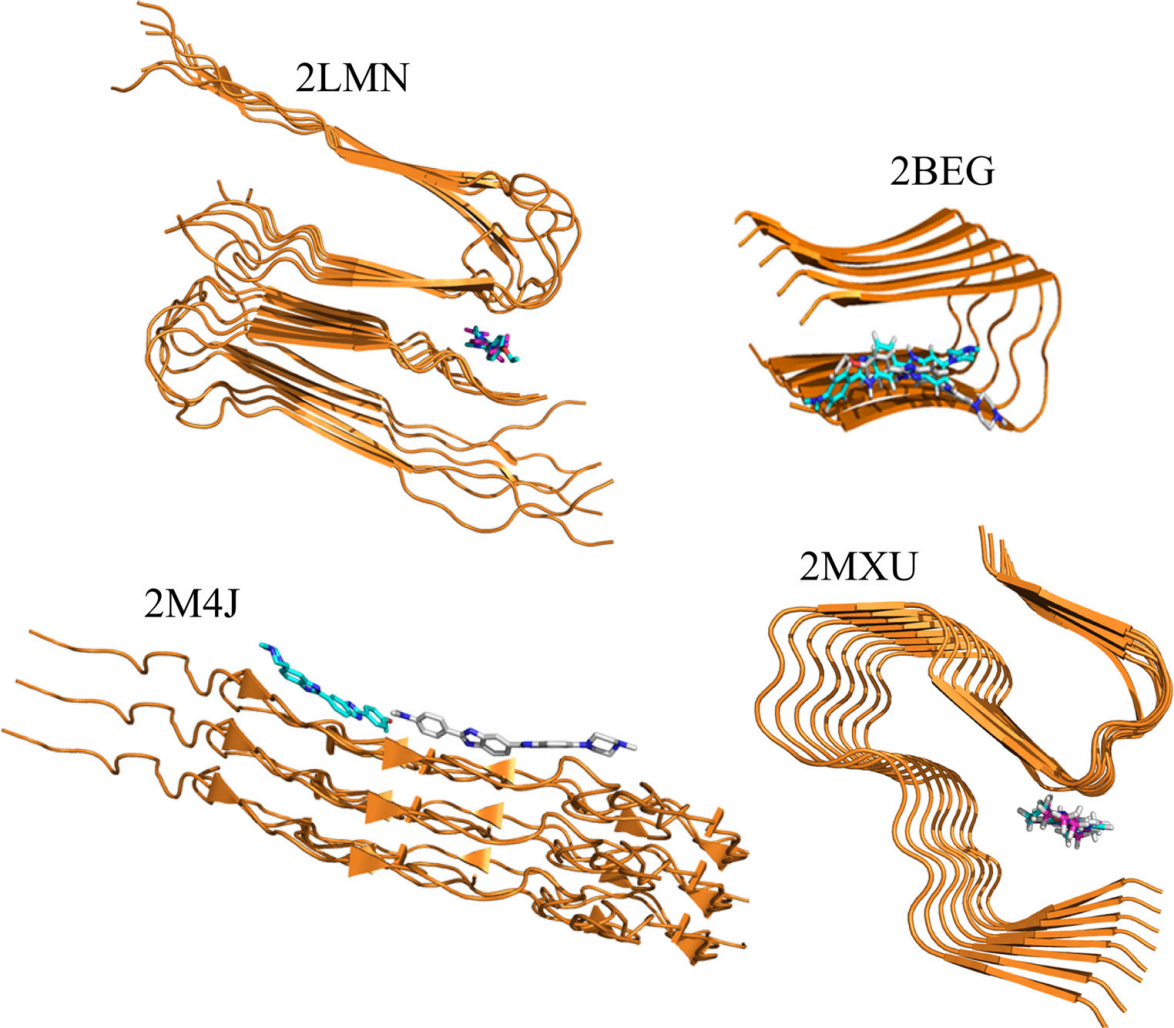
Binding poses of dyes Hoechst 34580 and Hoechst 33342 in 2LM, 2BEG, 2M4J and 2MXU. The structures were obtained by the docking method

Because we succeeded to purchase DNA dyes Hoechst 34580 (CID: 448202) and Hoechst 33342 (CID: 1464) for in vitro experiment, we consider them in more detail. Their binding poses in 2LM, 2BEG, 2M4J and 2MXU are shown in Fig. 3. Except full length fibril 9Aβ1-40 (2M4J), derived from a human patient, Hoechst 34580 and Hoechst 33342 have nearly the same binding positions in the remaining targets. The DNA dyes are located between two layers near the turn region of 2LMN, inside 2MXU, partially inside 2BEG and outside 2M4J fibrils (Fig. 3). Moreover, for a given target their binding energies are very close to each other (Tables S3–S5). This also holds for 2M4J, where two ligands bind to different places but Δ*E*_bind_ = −7.27 and −7.15 kcal/mol for Hoechst 34580 and Hoechst 33342, respectively. These results, as shown below, are consistent with the fact that they also have close rupture forces obtained by MD simulations and close binding free energies. The minor difference in their binding affinity is presumably due to high structural similarity (Fig. 2).

**Fig. 4:**
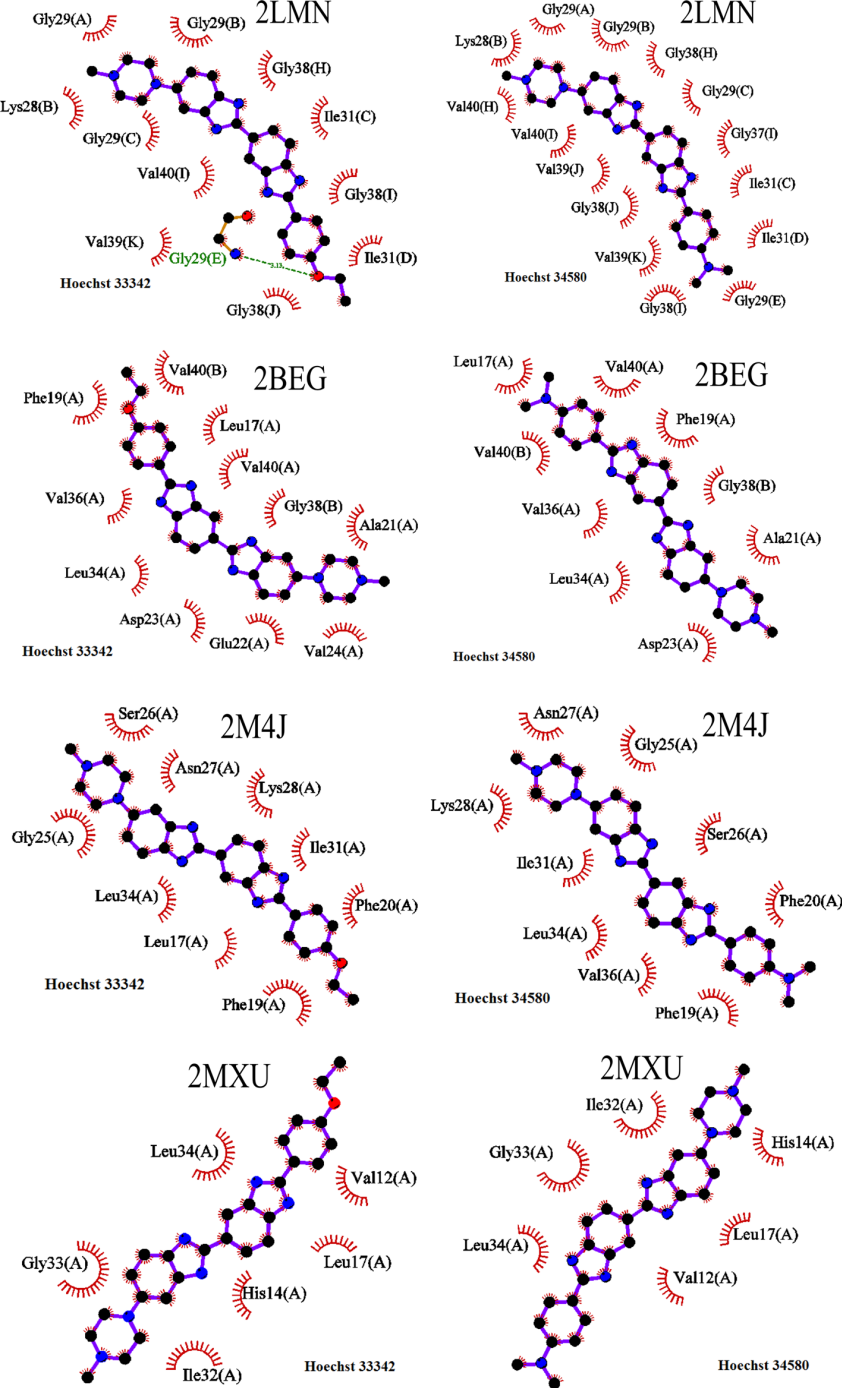
A HBs (*green dashed line*) and side chain non-bonded contacts (represented by an arc with spokes radiating towards the ligand atoms they contact) between four fibrils and DNA dyes Hoechst 34580 and Hoechst 33342. The plot was prepared using LigPlot + version 1.4.4 [57]

In docking, Hoechst 33342 forms one hydrogen bond (HB) with 2LMN, but none HB was found for 2BEG, 2MXU and 2M4J, while Hoechst 34580 does not have hydrogen binding with four targets (Fig. 4). Because the binding affinity of these compounds is high the poor HB networks indicate that the number HBs alone is not sufficient enough to describe the binding affinity of these complexes.

For 2LMN, Hoechst 33342 has 11 side chain (SC) contacts with residues Val39(K), Gly38(J), Ile31(D), Gly38(I), Val40(I), Gly29(C), Ile31(C), Gly38(H), Lys28(B), Gly29(B) and Gly29(A), whereas Hoechst 34580 forms 15 SC contacts with Val39(K), Gly29(E), Ile31(D), Gly38(I), Ile31(C), Gly37(I), Gly29(C), Gly38(H), Gly29(B), Gly29(A), Val40(H), Lys28(B), Val40(I), Val39(J) and Gly38(J) (Fig. 4). Here letters in parentheses refer to chains shown in Figure S1 in SI. In 2BEG, Hoechst 33342 has 11 SC contacts with residues Leu17(A), Gly38(B), Ala21(A), Asp23(A), Val24(A), Leu34(A), Glu22(A), Val36(A), Val40(A), Phe19(A), Val40(B) and Hoechst 34580 forms 9 contacts with residues Leu34(A), Asp23(A), Ala21(A), Val36(A), Phe19(A), Gly38(B), Val40(B), Val40(A), Leu17(A) (Fig. 4). Both DNA dyes have 9 SC contacts with 2M4J including the one with the charged residue Lys28(A) (Fig. 4). Because both ligands at the same place in 2MXU, they have 6 SC contacts with residues Val12(A), Leu17(A), His14(A), Gly33(A), Ile32(A), Leu34(A).

It should be noted that Hoechst 34580 and Hoechst 33342 prefer to stay next to hydrophobic residues of four targets (Fig. 4). In 2LMN and 2M4J they have only one contact with the positively charged residue Lys28 leading to the dominant role of the vdW interaction over the electrostatic interaction in stabilization of fibril-ligand complexes (see below).

#### Blood brain barrier

Using PreADME, we have calculated log(BB) for ligands revealed by the docking method as the top hits. Choosing only those ligands which have log(BB) > 0 one can further reduce the set to 36 ligands for 2LMN, 11 ligands for 2BEG and 13 ligands for 2MXU (Fig. 1). Hoechst 34580 and Hoechst 33342 are capable to easily cross BBB having log(BB) = 0.73 and 0.67, respectively.

#### Steered molecular dynamics

**Fig. 5:**
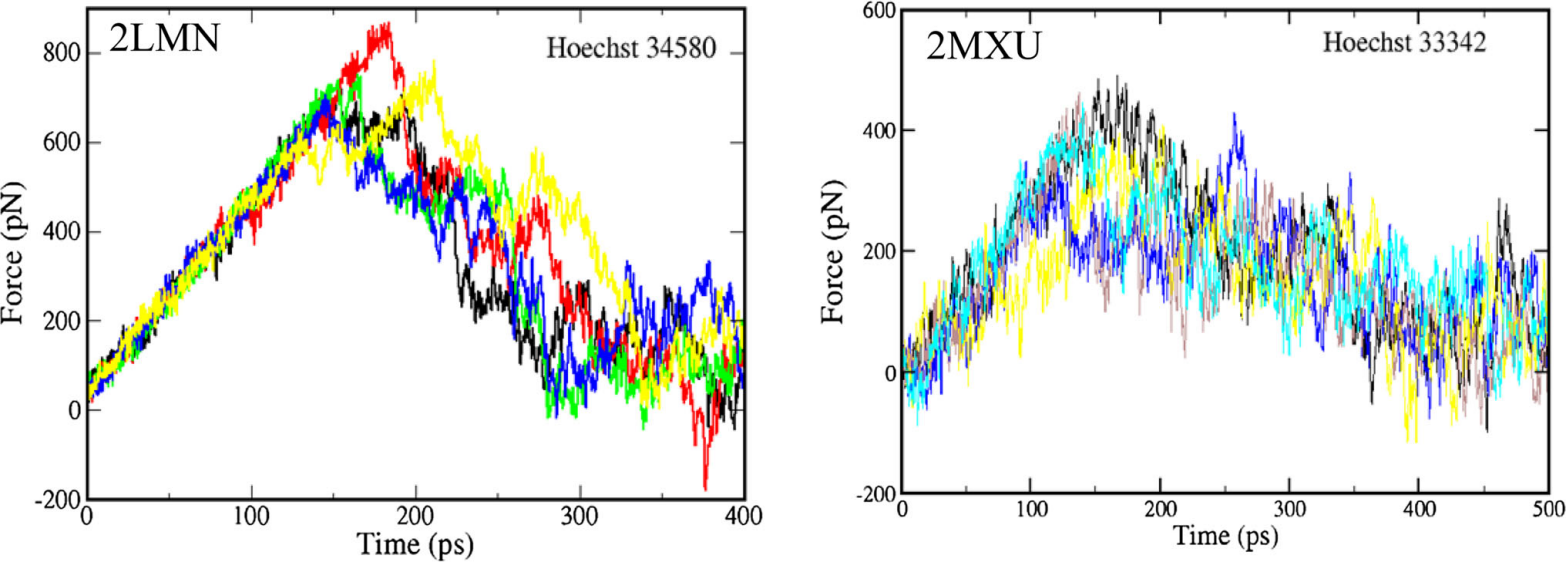
Force-time profiles obtained by the SMD method in five independent trajectories for 2LMN-Hoechst 34580 and 2MXU- Hoechst 33342 complexes

Using the Caver 3.0 [52] one can obtain several possible pulling directions but the easiest pathway with the lowest rupture force *F*_max_ [50] was chosen. Two representative optimal directions are shown in Figure S4 for ligands inside receptor 2MXU and between layers of 2LMN. For each ligand five independent SMD runs were performed and the results were averaged over all trajectories. Typical force–time curves are presented in Fig. 5 showing the sensibility of rupture force on SMD runs.

For receptor 2MXU, the SMD method was applied to study the binding affinity of 13 top leads including two DNA dyes. The SMD and docking results are shown in Table S3 together with ligand structures. The ranking of binding affinities based on docking energies is different from that predicted by SMD. Hoechst 34580 is champion in SMD but it is fourth in docking. SMD predicts that among 13 top hits compound CID 447767 is the weakest binder having the lowest rupture force. Consistent with the docking results, Hoechst 34580 and Hoechst 33342 have nearly the same rupture force *F*_max_ (Table S3).

For receptor 2LMN, keeping only those ligands which have *F*_max_ exceeding 470 pN the set of 36 ligands was reduced to 15 compounds as the top leads for AD including Hoechst 34580 and Hoechst 33342 (Table S4). As expected the ranking by docking binding energy is different from the SMD one. Compound CID 5327177 which is strongest in docking becomes seventh in SMD, while CID 6083166 twelfth in docking is first by SMD. Having applied the SMD method to 11 top leads bound with 2BEG we obtained the results shown in Table S5. In SMD Hoechst 34580 and Hoechst 33342 are at positions 10 and 7, respectively.

As evident from Tables S3-S5, in addition to Hoechst 34580 and Hoechst 33342, compound CID 447767 tightly binds to three models of Aβ40 and Aβ42 fibril. Thus, we predict that 3 compounds can interfere with both Aβ40 and Aβ42 aggregation.

#### MM-PBSA results

To make a direct comparison with experiments, we used the MM-PBSA method to compute Δ*G*_bind_ of Hoechst 34580 and Hoechst 33342 using Eq. (1). The conformations obtained in the best docking mode (Fig. 3) were used as starting conformations for all-atom MD simulation. For each fibril-ligand complex we performed four 100-150 ns MD runs starting from the same initial conformation but different random seed numbers. From the time dependence of C_α_ root mean square displacement (RMSD) of Aβ fibril, it is evident that all complexes reach equilibrium after about 40–100 ns (Figures S5 and S6 in SI). Snapshots stored every 20 ps in equilibrium were used to estimate the binding free energy given by Eq. (1).

For all studied targets, the vdW interaction dominates over the electrostatic interaction in directing ligand binding to Aβ because both Δ*G*_elec_ and Δ*G*_vdW_ are negative but the absolute value of Δ*G*_vdW_ is larger than Δ*G*_elec_ (Table 1). The reason behind this is that, as mentioned above, the DNA dyes locate rather close to hydrophobic residues than to the charged ones (Fig. 4). Due to geometrical similarity of Hoechst 34580 and Hoechst 33342 the entropic contributions are nearly the same for all complexes (Table 1). For a given target including 2BEG, 2M4J and 2MXU the binding free energies of two ligands are the same within error bars. The situation is different for 2LMN where Hoechst 34580 shows binding affinity higher than that of Hoechst 33342. The departure of this target from others is presumably caused by the fact that the DNA dyes are positioned next to the charged residue Lys28(B) (Fig. 4) in 2LMN but it is not the case for other targets where the ligands are surrounded by non-charged residues. This is also supported by the pronounced difference in electrostatic contributions of Hoechst 34580 and Hoechst 33342 to the binding propensity to 2LMN (Table 1).

**Table 1:** Binding free energy (kcal/mol), obtained by MM-PBSA method, for Hoechst 34580 and Hoechst 33342 using the AMBER-f99SB-ILDN force field

Receptor	Complex	$\Delta {\text{G}}_{\text{elec}}$	$\Delta {\text{G}}_{\text{vdW}}$	$\Delta {\text{G}}_{\text{PB}}$	$\Delta {\text{G}}_{\text{sur}}$	${\text{T}}\Delta {\text{S}}$	$\Delta {\text{G}}_{\text{bind}}$
2LMN	Hoechst 34580	−14.94	−61.94	31.56	−7.47	−25.87	−26.93 ± 1.89
	Hoechst 33342	−8.28	−58.66	32.47	−5.60	−23.30	−16.77 ± 2.43
2M4J	Hoechst 34580	−10.66	−41.19	23.26	−4.56	−23.07	−10.07 ± 6.24
	Hoechst 33342	−8.00	−36.33	14.19	−5.76	−21.81	−14.08 ± 2.27
2BEG	Hoechst 34580	−3.89	−49.78	15.51	−7.11	−21.61	−23.66 ± 4.47
	Hoechst 33342	−10.92	−51.31	22.68	−5.43	−22.91	−22.08 ± 5.73
2MXU	Hoechst 34580	−4.65	−65.32	29.06	−5.89	−23.21	−23.59 ± 1.12
	Hoechst 33342	−4.51	−52.23	21.43	−8.38	−23.11	−20.57 ± 4.37

Results were averaged over 4 MD trajectories

The difference in binding free energies of two DNA dyes to 2M4J was also seen (Table 1) due to contact with positively charged residue Lys28, but it is not as pronounced as in 2LMN because the ligands are positioned outside fibril (Fig. 3). Finally, Hoechst 34580 and Hoechst 33342 show the lowest binding affinity to 2M4J (Table 1) presumably because they are not located inside fibril. However, this is valid for a single fibril. There is also a possibility that DNA dyes interfere with the association between fibrils before acting at the individual fibril level. Then the interaction with 2M4J may get enhanced because they can be considered as located inside fibrils. This issue calls for further investigation.

In agreement with the docking simulations, SMD and experimental results (see below), within the error bars Δ*G*_bind_ of both dyes are the same for Aβ42 fibril. Having Δ*G*_bind_ < −10 kcal/mol for all targets, Hoechst 34580 and Hoechst 33342 are expected to block the Aβ40 and Aβ42 aggregation.

In order to shed more light on the binding mechanisms, we divided Hoechst 34580 and Hoechst 33342 into five blocks (Figure S7 in SI). The first four blocks are similar and the difference is in blocks 5 with the last atoms 55–62 for Hoechst 33342 and 55–63 for Hoechst 34580.

The contributions from 5 blocks to the vdW interactions depend on targets but for a given target they are similar for two dyes (Tables S6–S9 and Figure S8). The contribution of block 5 is less important than other blocks because it has the least number of atoms (8 and 9 atoms for Hoechst 33342 and Hoechst 34580, respectively). Block 4 contributes to the vdW interactions less than blocks 1–3 and this holds for all fibril models (Tables S6–S9).

Although contributions of individual blocks to the electrostatic interactions are highly diverse, for all targets the difference between Hoechst 34580 and Hoechst 33342 is most pronounced for block 5 (Tables S6–S9 and Figure S9). In 2LMN the Coulomb interaction between block 5 and Hoechst 33342 is 2.78 kcal/mol but it is −24.49 kcal/mol for Hoechst 34580 (Table S6). Due to the proximity with block 5 the difference between two ligands in electrostatic interactions of block 4 is more than blocks 1–3.

Atoms 26, 29, 30, 37, 42 and 43 play a crucial role in electrostatic interaction of Hoechst 34580 with 2LMN having Δ*E*_elec_ < −30 kcal/mol (Figure S9), while for Hoechst 33342 atoms 24, 26, 29, 30, 38, 42, 43 and 56 are vital. In 2BEG atoms 26, 29, 30, 37, 42 and 43 make a major contribution for Hoechst 33342, whereas atoms 24, 26, 29, 30, 38, 42 and 43 of Hoechst 34580 are dominating. Atoms 29, 30, 42 and 43 drive the electrostatic interaction of two dyes with all four targets including (Figure S9).

Block 3 of Hoechst 33342 is superior in Coulomb interaction with 2LMN, 2M4J and 2MXU while for 2BEG block 2 is the most important (Tables S6–S9). For Hoechst 34580 block 2 tightly binds to 2LMN and 2M4J, but for 2BEG and 2MXU block 1 is dominating.

The contributions of individual blocks to the total interaction (vdW and electrostatic) are shown in Tables S6–S9 and Figure S10. For Hoechst 33342 block 3 is dominating for targets 2LMN, 2M4J and 2MXU but in 2BEG block 2 is the most prominent. Block 5 of Hoechst 34580 is the most important for 2LMN and 2M4J, while block 1 and 3 have the highest binding propensity to 2BEG and 2MXU.

Upon binding of Hoechst 33342 and Hoechst 34580 the solvent accessible surface area (SASA) of all complexes shrinks leading to the negative value of Δ*G*_sur_ (Table 1). The impact of the dyes depends on targets that Hoechst 34580 changes SASA of 2LMN and 2BEG to a larger extent than Hoechst 33342, while the opposite effect occurs in 2M4J and 2MXU.

### Experimental results

#### Inhibition of Aβ42 aggregation by Hoechst 34580 and Hoechst 33342

**Fig. 6:**
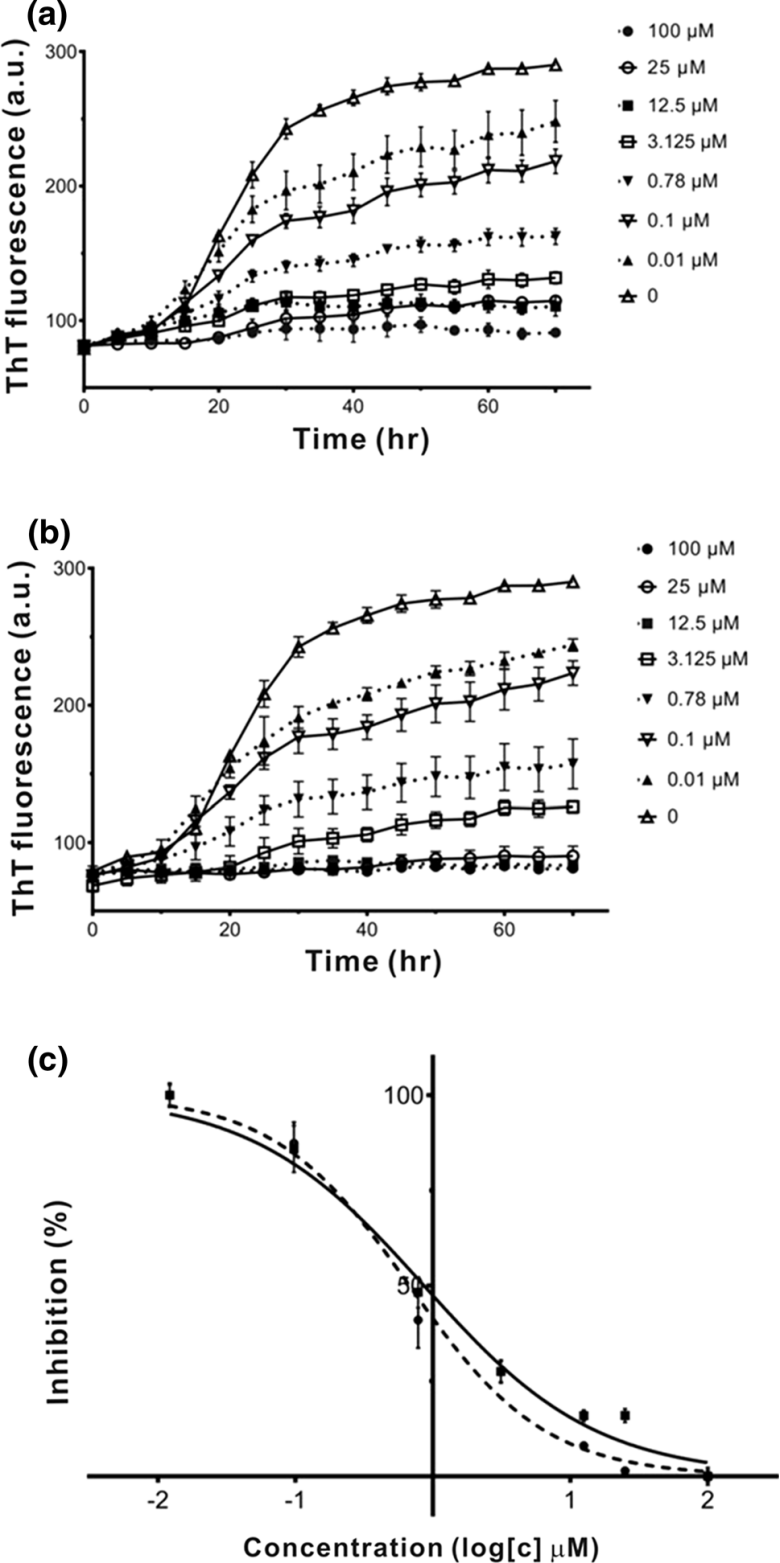
Fibrillization kinetics of Aβ42 incubated with and without Hoechst 34580 or Hoechst 33342. **a** Various concentrations (0.01–100 μM) of Hoechst 34580 was incubated with 50 μM Aβ42 at 37 °C for 70 h; **b** Various concentrations (0.01–100 μM) of Hoechst 33342 was incubated with 50 μM Aβ42 at 37 °C for 70 h; **c** Variation in ThT fluorescence intensity as a function of Hoechst 34580 (*solid line*) and Hoechst 33342 (*dashed line*). The data were subtracted to background from compound alone. Data were analyzed using GraphPad Prism to obtain IC_50_ values using log (inhibitor) versus normalized response-variable slope. Dose–response curves showed fractional binding of 5 μM ThT to 50 μM Aβ42 fibrils in the presence of Hoechst 34580 or Hoechst 33342, respectively

To evaluate the inhibitory effect of these compounds against Aβ42 aggregation, a Thioflavin T (ThT) assay was performed to monitor Aβ fibril formation while respectively co-incubating with various concentrations of the compounds (Fig. 6a, b). When ThT bound to cross β-sheets of fibrils, its emission fluorescence intensity could be measured which indicates the quantity of relative fibril formation. 50 μM Aβ42 solutions co-incubated with 100, 25, 12.5, 3.125, 0.78, and 0.1, 0.01 μM Hoechst 34580 or Hoechst 33342 at 37 °C for 70 h. We found that these compounds could inhibit the aggregation of Aβ42 in a dose-dependent manner. And then we examined the half-maximal concentration (IC_50_) required (Fig. 6c) to compare the potency of these three compounds. The IC_50_ was obtained by measuring the concentration of Hoechst 34580 and Hoechst 33342 respectively while maintaining the Aβ42 concentration which gave 0.86 ± 0.05 μM for Hoechst 34580 and 0.68 ± 0.05 μM for Hoechst 33342.

To compare the experimental results with the simulation results one cannot use the binding free energy obtained for 2BEG (5Aβ17-42) because this structure is based on H/D exchange and therefore, is not an experimentally observed structure. However, in vitro results may be compared with the results obtained for the solid state NMR structure 8Aβ11-42 (2MXU). With the equation Δ*G*_bind_ = *RT* ln(IC50), where gas constant $R = 1.987 \times 10^{ - 3} {\text{kcal K}}^{ - 1} {\text{mol}}^{ - 1}$, *T* = 300 K and inhibition constant IC50 is measured in mol, a binding constant of 1 nM corresponds to Δ*G*_bind_ ≈ −12.8 kcal/mol. A change in IC50 of one order of magnitude results in a change in the binding free energy of 1.4 kcal/mol. Therefore, the calculated values of Δ*G*_bind_ for 2MXU (Table 1) imply that IC50 of both DNA dyes could be much less than 1 pM. They are also too far away from the experimentally measured value. The reason behind the discrepancy between theory and experiment is that it is very hard to match the calculated absolute binding free energy with experiments as it depends not only on force fields [53] but also on theoretical methods [59]. However, theoretically estimated binding free energies are presumably useful for ranking binding affinities [59]. This is also evident from our results that, in agreement with experiments, within the error bars Hoechst 34580 and Hoechst 33342 have the same binding free energy (Table 1). Therefore, our theoretical results on Δ*G*_bind_ are useful for prediction of binding affinity ranking rather than for a direct comparison with experimentally measured inhibition constants.

## Conclusion

Using the multi-step virtual screening we have predicted several compounds as potential drugs for AD. The ability of Hoechst 34580 and Hoechst 33342 in blocking Aβ aggregation was confirmed also by in vitro experiments. These compounds are located next to hydrophobic residues of Aβ peptides. The vdW interaction is dominating over the electrostatic interaction in binding propensity. The QSAR analysis showed that Hoechst 34580 and Hoechst 33342 can easily cross BBB having log(BB) greater than 0.5. Because these DNA dyes are known to be not cytotoxic they are recommended for further in vivo studies.

## Future directions

In collaboration with experimentalists, our future work will be focused on in vivo study of the impact of DNA dyes Hoechst 34580 and Hoechst 33342 on Aβ aggregation. We plan also to search for new potential inhibitors from other large databases.

## Electronic supplementary material

Below is the link to the electronic supplementary material.

Atomic names, types, masses and charges used in the MD simulation of Hoechst 33342 and Hoechst 34580 by AMBER-f99SB-ILDN force field are listed in Table S1 and S2. Binding affinities and ligand ranking obtained by docking and SMD simulations are shown in Table S3–S5. Contributions of individual blocks to the interaction energy of DNA dyes with Aβ fibrils are presented in Tables S6-S9. Figure S1 shows the PDB structures of fibril 12Aβ_9–40_ (PDB ID 2LMN), 5Aβ_17–42_ (PDB ID 2BEG), 9Aβ_1–40_ (PDB ID 2M4J), and 8Aβ_11–42_ (PDB ID 2MXU). Figure S2 depicts the population of binding energies of 5372 ligands to 2MXU, 2LMN and 2BEG. Figure S3 show binding positions of top hits obtained by docking simulation for 2LMN, 2BEG and 2MXU. Figures S4 and S5 showed the time dependence of RMSD for four targets in complex with two DNA. Figures S6–S9 depict per-atom distribution of vdW and electrostatic interactions of 5 blocks of Hoechst 34580 and Hoechst 33342 with four fibril models. (PDF 1414 kb)
