# Supplementary material for: Discovery of DNA dyes Hoechst 34580 and 33342 as good candidates for inhibiting amyloid beta formation: in silico and in vitro study
Source: J Comput Aided Mol Des. 2016 Aug 10;30(8):639–50. doi: 10.1007/s10822-016-9932-1 (PMC5021751; doi:10.1007/s10822-016-9932-1)
Supplement: Supplementary file 1 — Atomic names, types, masses and charges used in the MD simulation of Hoechst 33342 and Hoechst 34580 by AMBER-f99SB-ILDN force field are listed in Table S1 and S2. Binding affinities and ligand ranking obtained by docking and SMD simulations are shown in Table S3–S5. Contributions of individual blocks to the interaction energy of DNA dyes with Aβ fibrils are presented in Tables S6-S9. Figure S1 shows the PDB structures of fibril 12Aβ9–40 (PDB ID 2LMN), 5Aβ17–42 (PDB ID 2BEG), 9Aβ1–40 (PDB ID 2M4J), and 8Aβ11–42 (PDB ID 2MXU). Figure S2 depicts the population of binding energies of 5372 ligands to 2MXU, 2LMN and 2BEG. Figure S3 show binding positions of top hits obtained by docking simulation for 2LMN, 2BEG and 2MXU. Figures S4 and S5 showed the time dependence of RMSD for four targets in complex with two DNA. Figures S6–S9 depict per-atom distribution of vdW and electrostatic interactions of 5 blocks of Hoechst 34580 and Hoechst 33342 with four fibril models. (PDF 1414 kb) [file 10822_2016_9932_MOESM1_ESM.pdf]

Discovery of DNA dyes Hoechst 34580 and 33342 as good candidates for inhibiting amyloid beta formation: *in silico* and *in vitro* study

Nguyen Quoc Thai[1,2,3], Ning-Hsuan Tseng[4], Mui Thi Vu[1], Tin Trung Nguyen[1], Huynh Quang Linh[2], Chin-Kun Hu[5,6,7 ‡], Yun-Ru Chen[4,‡], and Mai Suan Li[1,8,‡].

[1]Institute for Computational Science and Technology, SBI building, Quang Trung Software City, Tan Chanh Hiep Ward, District 12, Ho Chi Minh City, Vietnam

[2]Biomedical Engineering Department, University of Technology -VNU HCM, 268 Ly Thuong Kiet Str., Distr. 10, Ho Chi Minh City, Vietnam

[3]Division of Theoretical Physics, Dong Thap University, 783 Pham Huu Lau Street, Ward 6, Cao Lanh City, Dong Thap, Vietnam

[4]Genomics Research Center, Academia Sinica, Academia Rd., Sec. 2, Nankang Dist., Taipei 115, Taiwan

[5]Institute of Physics, Academia Sinica, 128 Academia Road Section 2, Taipei 11529, Taiwan

[6]National Center for Theoretical Sciences, National Tsing Hua University, 101 Kuang-Fu Road Section 2, Hsinch 30013, Taiwan.

[7]Business School, University of Shanghai for Science and Technology, 334 Jun Gong Road, Shanghai 200093, China

[8]Institute of Physics, Polish Academy of Sciences, Al. Lotnikow 32/46, 02-668 Warsaw, Poland

‡ Email: masli@ifpan.edu.pl; huck@phys.sinic.edu.tw; yrchen@gate.sinica.edu.tw

SUPPORTING INFORMATION

Table S1. Names, types atoms, masses and charges of atoms used in the simulation of Hoechst 33342 by AMBER-f99SB-ILDN force field.

Nr	Type	Atom	Charge	Mass
1	h1	H1	0.057031	1.00800
2	c3	C1	-0.07282	12.0100
3	h1	H2	0.057031	1.00800
4	h1	H3	0.057031	1.00800
5	n3	N1	-0.25248	14.0100
6	c3	C2	-0.08494	12.0100
7	h1	H4	0.078889	1.00800
8	h1	H5	0.078889	1.00800
9	c3	C3	-0.09717	12.0100
10	h1	H6	0.094148	1.00800
11	h1	H7	0.094148	1.00800
12	nh	N2	-0.16525	14.0100
13	c3	C4	-0.09717	12.0100
14	h1	H8	0.094148	1.00800
15	h1	H9	0.094148	1.00800
16	c3	C5	-0.08494	12.0100
17	h1	H10	0.078889	1.00800
18	h1	H11	0.078889	1.00800
19	ca	C6	0.055009	12.01000
20	ca	C7	-0.06567	12.0100
21	ha	H12	0.088317	1.00800
22	ca	C8	-0.288207	12.0100
23	ha	H13	0.158276	1.00800
24	ca	C9	0.310627	12.01000
25	ca	C10	-0.29439	12.0100
26	ha	H14	0.167321	1.00800
27	ca	C11	0.080638	12.01000
28	na	N3	-0.345553	14.01000
29	hn	H15	0.306297	1.00800
30	cc	C12	0.355692	12.01000
31	nd	N4	-0.521200	14.01000
32	ca	C13	-0.04935	12.0100
33	ca	C14	-0.06837	12.0100
34	ha	H16	0.101583	1.00800
35	ca	C15	-0.29154	12.0100
36	ha	H17	0.147214	1.00800

37	ca	C16	0.177816	12.01000
38	ca	C17	0.225023	12.01000
39	ca	C18	-0.20618	12.0100
40	ha	H18	0.141074	1.00800
41	na	N5	-0.437476	14.01000
42	hn	H19	0.329237	1.00800
43	cc	C19	0.423461	12.01000
44	nd	N6	-0.480673	14.01000
45	ca	C20	-0.12608	12.0100
46	ca	C21	-0.10764	12.0100
47	ha	H20	0.109542	1.00800
48	ca	C22	-0.10764	12.0100
49	ha	H21	0.109542	1.00800
50	ca	C23	-0.10173	12.0100
51	ha	H22	0.098267	1.00800
52	ca	C24	0.125718	12.01000
53	os	O1	-0.260954	16.00000
54	c3	C25	0.183995	12.01000
55	h1	H23	0.030340	1.00800
56	h1	H24	0.030340	1.00800
57	ca	C26	-0.10173	12.0100
58	ha	H25	0.098267	1.00800
59	c3	C27	-0.17173	12.0100
60	hc	H26	0.054706	1.00800
61	hc	H27	0.054706	1.00800
62	hc	H28	0.054706	1.00800

Table S2. Names, types, masses and charges of atoms used in the simulation of Hoechst 34580 by AMBER-f99SB-ILDN force field.

Nr	Type	Atom	Charge	Mass
1	h1	H1	0.059036	1.00800
2	c3	C1	-0.08420	12.0100
3	h1	H2	0.059036	1.00800
4	h1	H3	0.059036	1.00800
5	n3	N1	-0.25914	14.0100
6	c3	C2	-0.07245	12.0100
7	h1	H4	0.077638	1.00800
8	h1	H5	0.077638	1.00800
9	c3	C3	-0.08691	12.0100
10	h1	H6	0.086434	1.00800

11	h1	H7	0.086434	1.00800
12	nh	N2	-0.15531	14.0100
13	c3	C4	-0.08691	12.0100
14	h1	H8	0.086434	1.00800
15	h1	H9	0.086434	1.00800
16	c3	C5	-0.07245	12.0100
17	h1	H10	0.077638	1.00800
18	h1	H11	0.077638	1.00800
19	ca	C6	0.025912	12.01000
20	ca	C7	-0.13977	12.0100
21	ha	H12	0.114647	1.00800
22	ca	C8	-0.215497	12.0100
23	ha	H13	0.140130	1.00800
24	ca	C9	-0.166246	12.0100
25	ha	H14	0.134020	1.00800
26	ca	C10	0.270678	12.01000
27	ca	C11	0.029060	12.01000
28	na	N3	-0.379194	14.01000
29	hn	H15	0.315536	1.00800
30	cc	C12	0.433418	12.01000
31	nd	N4	-0.538194	14.01000
32	ca	C13	-0.08226	12.0100
33	ca	C14	-0.06444	12.0100
34	ha	H16	0.096937	1.00800
35	ca	C15	-0.33671	12.0100
36	ha	H17	0.154683	1.00800
37	ca	C16	0.258496	12.01000
38	ca	C17	0.169677	12.01000
39	ca	C18	-0.18971	12.0100
40	ha	H18	0.136896	1.00800
41	na	N5	-0.473661	14.01000
42	hn	H19	0.332480	1.00800
43	cc	C19	0.408914	12.01000
44	nd	N6	-0.455372	14.01000
45	ca	C20	-0.09921	12.0100
46	ca	C21	-0.10275	12.0100
47	ha	H20	0.096433	1.00800
48	ca	C22	-0.12772	12.0100
49	ha	H21	0.121090	1.00800
50	ca	C23	-0.10275	12.0100
51	ha	H22	0.096433	1.00800
52	ca	C24	-0.12772	12.0100
53	ha	H23	0.121090	1.00800

54	ca	C25	0.000329	12.01000
55	nh	N7	-0.013424	14.01000
56	c3	C26	-0.17966	12.0100
57	h1	H24	0.083524	1.00800
58	h1	H25	0.083524	1.00800
59	h1	H26	0.083524	1.00800
60	c3	C27	-0.17966	12.0100
61	h1	H27	0.083524	1.00800
62	h1	H28	0.083524	1.00800
63	h1	H29	0.083524	1.00800

Table S3. Log(BB), binding energy E_{bind} and F_{max} obtained by the docking and SMD simulations for 13 top ligands bound to 2MXU. The first column shows the ranking by docking and SMD (in parentheses). The 3D structures of top hits are shown in the last column.

Ranking by Dock(SMD)	CID	Docking Ebind (kcal/mol)	F_{max}(pN)	log(BB)	Structure 3D
1(4)	1464 (Hoechst 33342)	-10.4	458	0.67	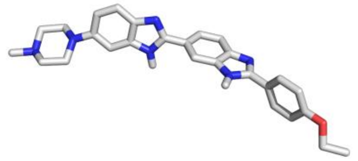
2(12)	5326397	-10.4	238	0.21	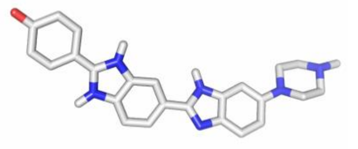
3(5)	444058	-10.0	449	0.53	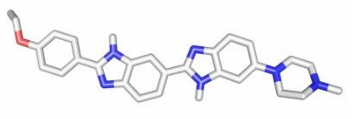
4(1)	448202(Hoechst 34580)	-9.8	498	0.73	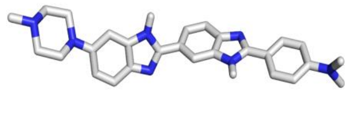
5(2)	448201	-9.8	482	0.84	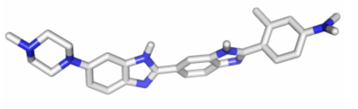
6(3)	442972	-9.7	473	0.82	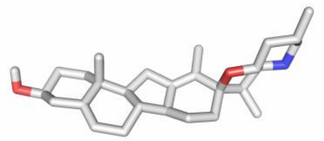

7(8)	447736	-9.7	395.46	0.04	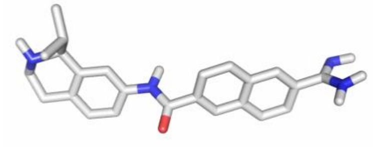
8(7)	11167118	-9.7	402	0.39	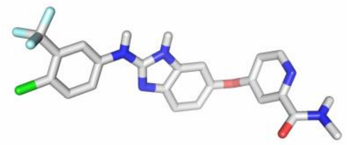
9(11)	11690363	-9.7	374	0.52	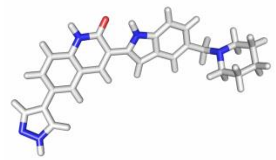
10(10)	447735	-9.6	377	0.10	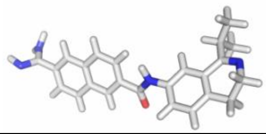
11(9)	6540268	-9.6	384	0.11	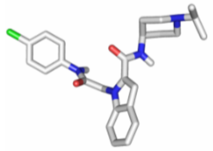
12(13)	447767	-9.3	157	0.89	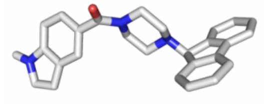
13(6)	9956146	-9.1	406	0.23	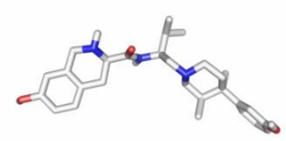

Table S4: Binding energy E_{bind}, obtained by docking, F_{max} and log(BB) of 15 top ligands revealed for receptor 2LMN by SMD simulation. The first column shows the ranking by docking and SMD (in parentheses). The 3D structures of top hits are shown in the last column.

Ranking by Dock(SMD)	CID	Docking E_{bind} (kcal/mol)	F_{max}(pN)	log(BB)	3D structure
1 (7)	5327177	-11.4	619	0.12	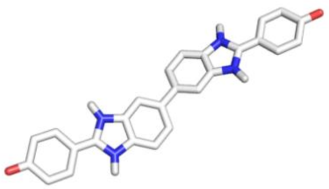

2 (5)	448202 (Hoechst 34580)	-10.6	682	0.74	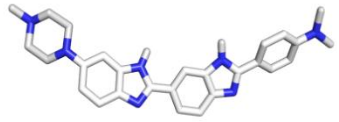
3 (9)	448201	-10.6	550	0.85	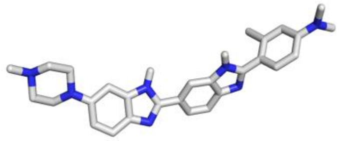
4 (2)	11545419	-10.4	695	0.84	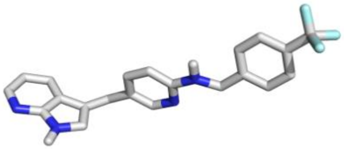
5 (8)	4369491	-10.4	556	0.18	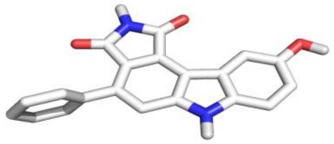
6 (3)	16062971	-10.3	692	0.09	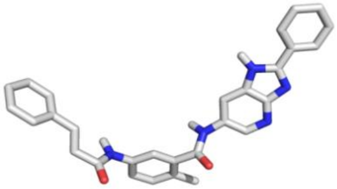
7 (6)	1464 (Hoechst 33342)	-10.3	664	0.67	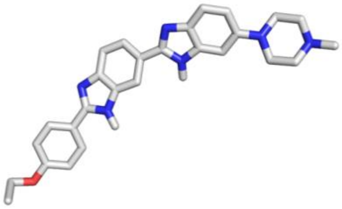
8 (10)	5327055	-10.3	536	0.16	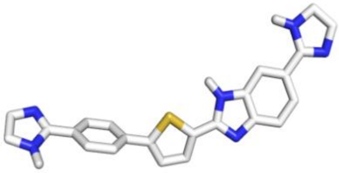
9 (11)	447767	-10.2	532	0.89	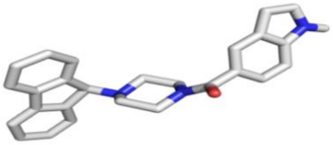

10 (13)	10297043	-10.2	513	0.78	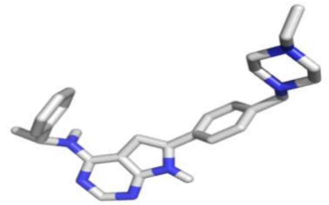
11 (14)	444980	-10.2	472	0.09	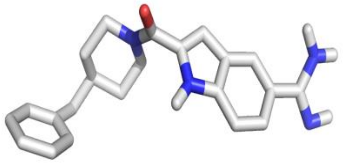
12 (1)	6083166	-10.10	700.59	0.16	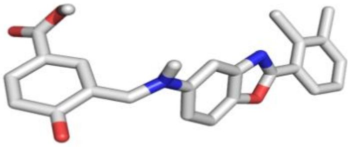
13 (12)	2399	-10.10	520.62	0.30	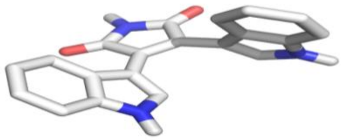
14 (4)	448793	-10.00	684.98	0.12	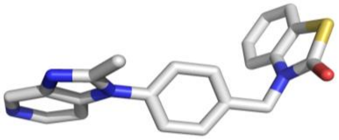
15 (15)	5330797	-10.00	474.25	0.60	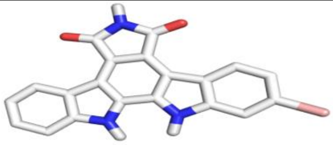

Table S5. Log(BB), binding energy E_{bind} and F_{max} obtained by the docking and SMD simulations for 11 top ligands bound to 2BEG. The first column shows the ranking by docking and SMD (in parentheses). The 3D structures of top hits are shown in the last column.

Ranking by Dock(SMD)	CID	Docking Ebind (kcal/mol)	F_{max}(pN)	log(BB)	Structure 3D
1(4)	16062971	-8.7	311	0.09	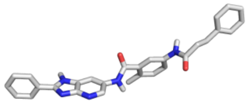

2(1)	11987705	-8.5	342	0.67	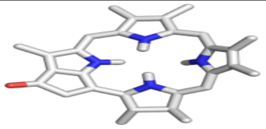
3(3)	5289319	-8.4	313	0	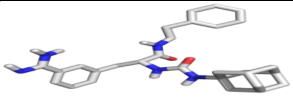
4(7)	1464 (Hoechst 33342)	-8.3	265	0.67	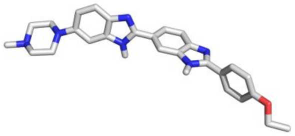
5(9)	444980	-8.1	240	0.53	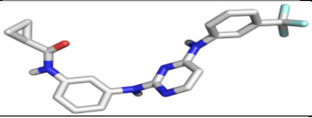
6(8)	5287621	-8.1	264	0.5	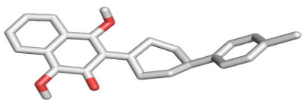
7(5)	9549303	-8.1	283	0.28	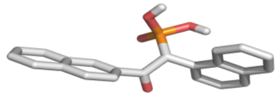
8(6)	447767	-8.0	275	0.89	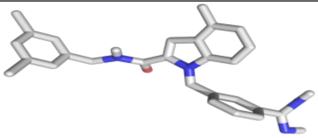
9(10)	448202(Hoechst 34580)	-8.0	204	0.74	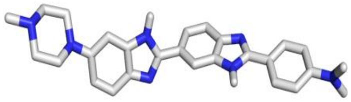
10(11)	11494412	-8.0	172	0.4	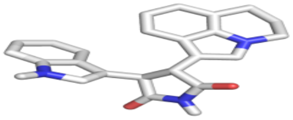
11(2)	6083166	-8.0	339	0.16	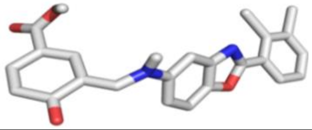

Table S6. The contributions of individual blocks to the interaction energy of Hoechst 33342 (and Hoechst 34580) with fibril 2LMN for the AMBER-f99SB-ILDN force field. The numbers in parentheses refer to Hoechst 34580.

Energy	Block 1	Block 2	Block 3	Block 4	Block 5
E_{vdW}	-14.27(-13.80)	-10.77(-15.34)	-14.04(-14.83)	-11.66(-11.15)	-7.89(-7.45)
E_{elec}	-2.91(-4.12)	-2.11(-7.16)	-4.81(0.90)	-1.05(20.03)	2.78(-24.49)
E_{vdw} + E_{elec}	-17.18(-17.92)	-12.88(-22.50)	-18.85(-13.93)	-12.71(8.88)	-5.11(-31.94)

Table S7. The contribution of individual blocks to the interaction energy of Hoechst 33342 (and Hoechst 34580) with fibril 2M4J for the force field AMBER-f99SB-ILDN. The numbers in parentheses refer to Hoechst 34580.

Energy	Block 1	Block 2	Block 3	Block 4	Block 5
E_{vdW}	-10.17(-9.08)	-9.30(-11.04)	-9.22(-11.2)	-5.74(-6.07)	-2.34(-3.78)
E_{elec}	-2.73 (-5.67)	-5.77(-13.1)	-7.28(10.01)	1.03(36.93)	7.03(-38.57)
E_{vdw} + E_{elec}	-12.90(-14.75)	-15.07(-24.14)	-16.50(-1.19)	-4.77(30.85)	5.37(-42.34)

Table S8. The contribution of individual blocks to the interaction energy of Hoechst 33342 (and Hoechst 34580) with fibril 2BEG for the force field AMBER-f99SB-ILDN. The numbers in parentheses are for Hoechst 34580.

Energy	Block 1	Block 2	Block 3	Block 4	Block 5
E_{vdW}	-15.17(-12.27)	-14.77(-12.84)	-12.25(-14.65)	-6.18(-7.89)	-2.95(-3.66)
E_{elec}	-0.92(-2.99)	-8.44(-1.68)	-3.73(1.81)	0.03(9.01)	2.2(-10.46)
E_{vdw} + E_{elec}	-16.01(-15.26)	-23.21(-14.52)	-15.98(-12.84)	-6.15(1.12)	-0.76(-14.12)

Table S9. The contribution of individual blocks to the interaction energy of Hoechst 33342 (and Hoechst 34580) with fibril 2MXU for the force field AMBER-f99SB-ILDN. The numbers in parentheses are for Hoechst 34580.

Energy	Block 1	Block 2	Block 3	Block 4	Block 5
E_{vdW}	-11.60(-14.94)	-13.06(-16.27)	-13.52(-17.27)	-10.20(-10.42)	-4.37 (-6.41)
E_{elec}	-0.74(-1.59)	-0.90(-0.85)	-2.47(-0.98)	-0.74(4.17)	0.39(-5.42)
$E_{vdw} + E_{elec}$	-12.34(-16.53)	-13.96(-17.12)	-15.99(-18.26)	-10.94(-6.25)	-3.98 (-11.83)

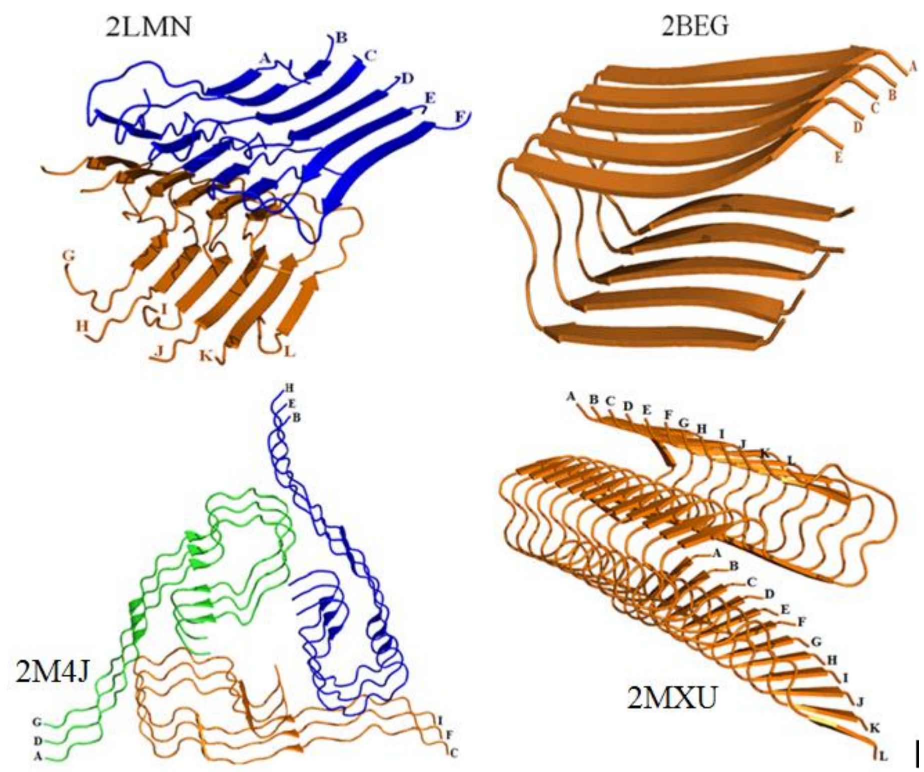

Figure S1. The PDB structure of Aβ40 fibrils (PDB ID: 2LMN and 2M4J) and Aβ42 fibrils (PDB ID 2BEG and 2MXU). Capital letters refer to polypeptide chains.

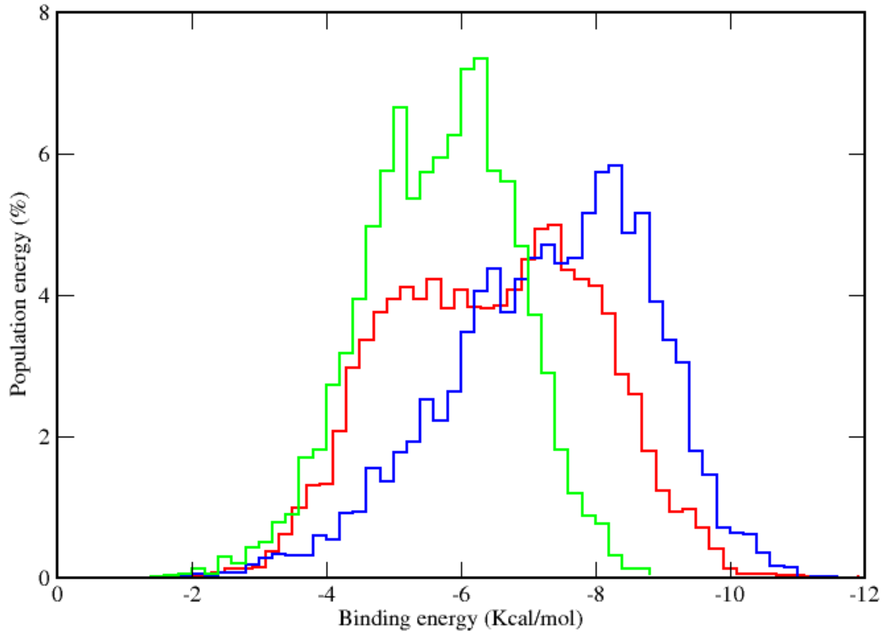

Figure S2. Population of binding energies of 5372 ligands to 2LMN (blue), 2MXU (red) and 2BEG (green). Results were obtained in the best docking mode.

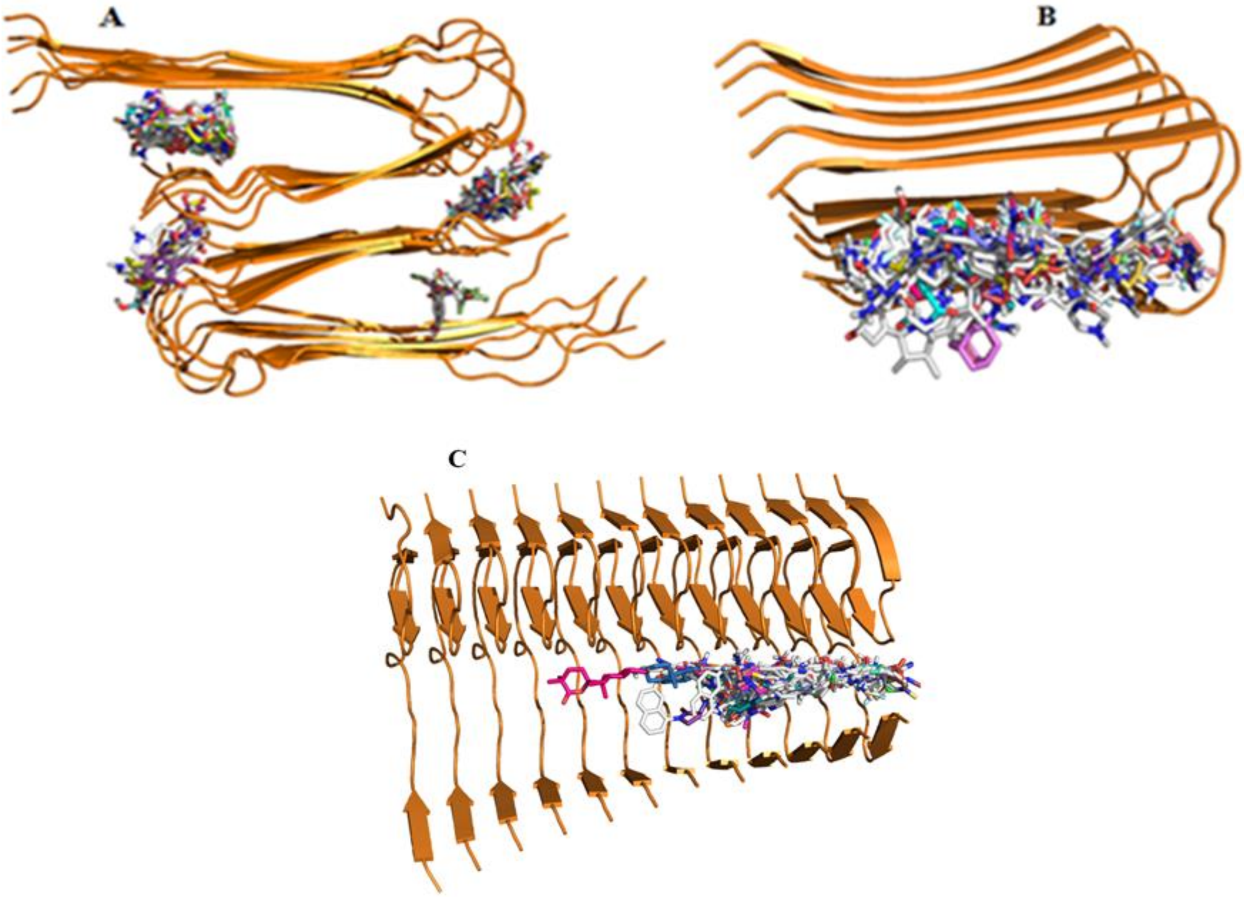

Figure S3. Binding positions of 96 compounds with the binding energy lower than -10 kcal/mol for 2LMN (A), 55 compounds with ΔE_{bind} < -8.0 kcal/mol for 2BEG (B), and 57 compounds with ΔE_{bind} < -9.0 kcal/mol for 2MXU (C). The structures were obtained in the best docking mode.

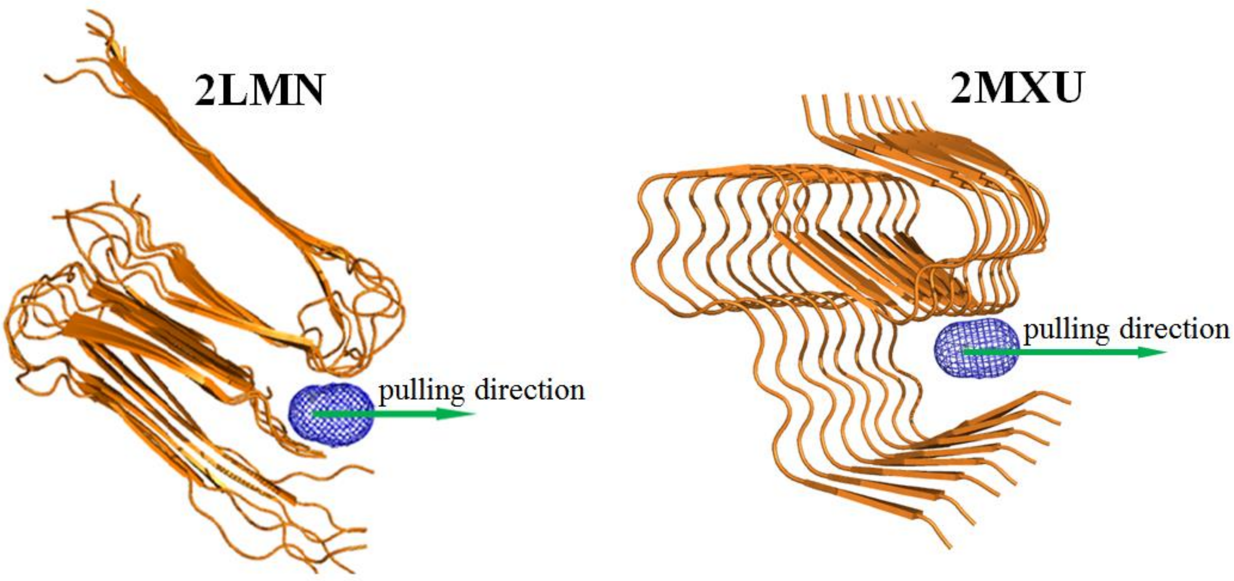

Figure S4. Optimal pulling directions revealed by the Caver 3.0 software for Hoechst 33342 and Hoechst 33342 to escape from 2LMN (left) and from 2MXU (right).

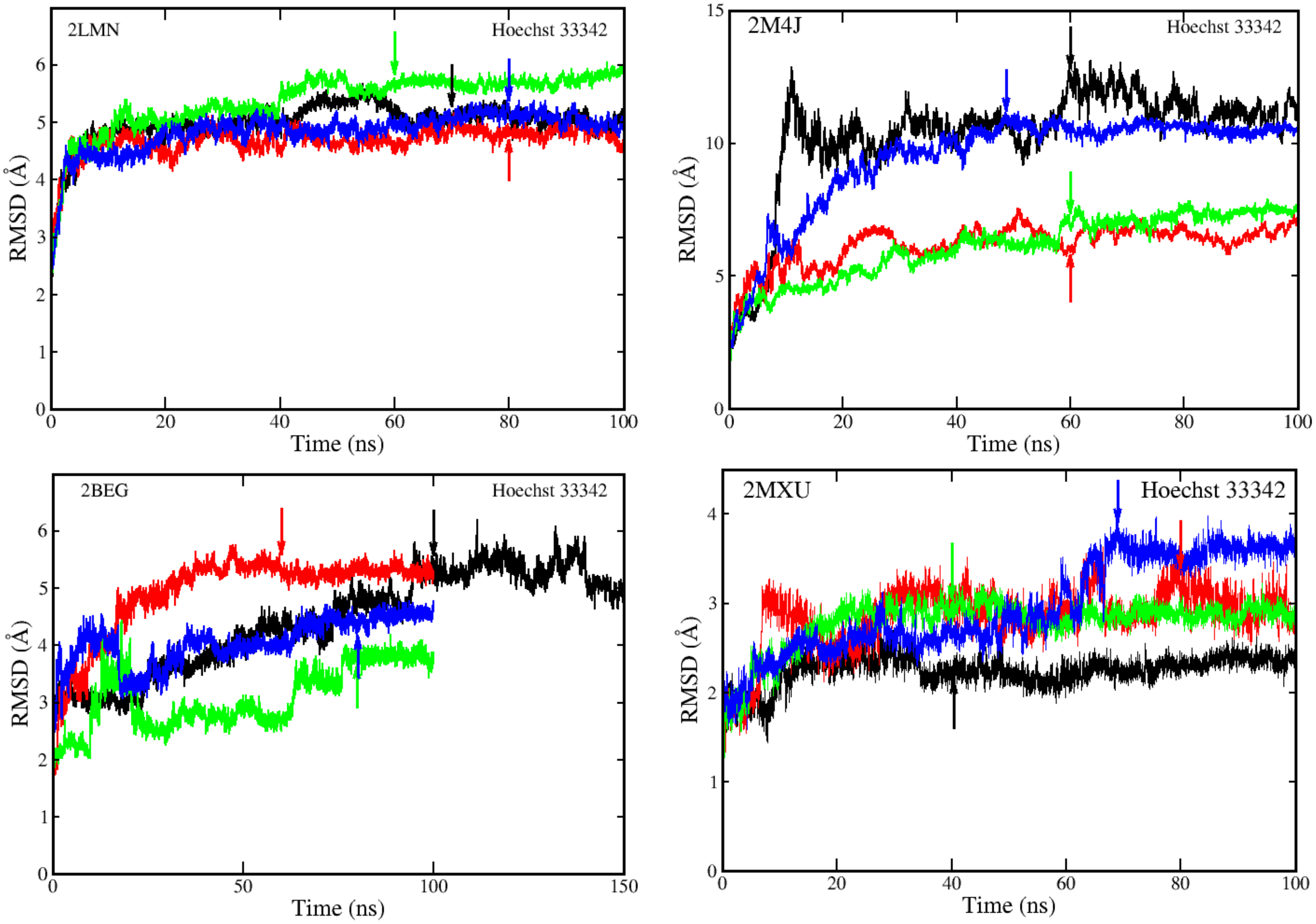

Figure S5. Time dependence of RMSD with respect to the initial structure for 2LMN, 2M4J, 2BEG and 2MXU in complex with Hoechst 33342 with the force field AMBER-f99SB-ILDN. Arrows refer to time when the systems reach equilibrium.

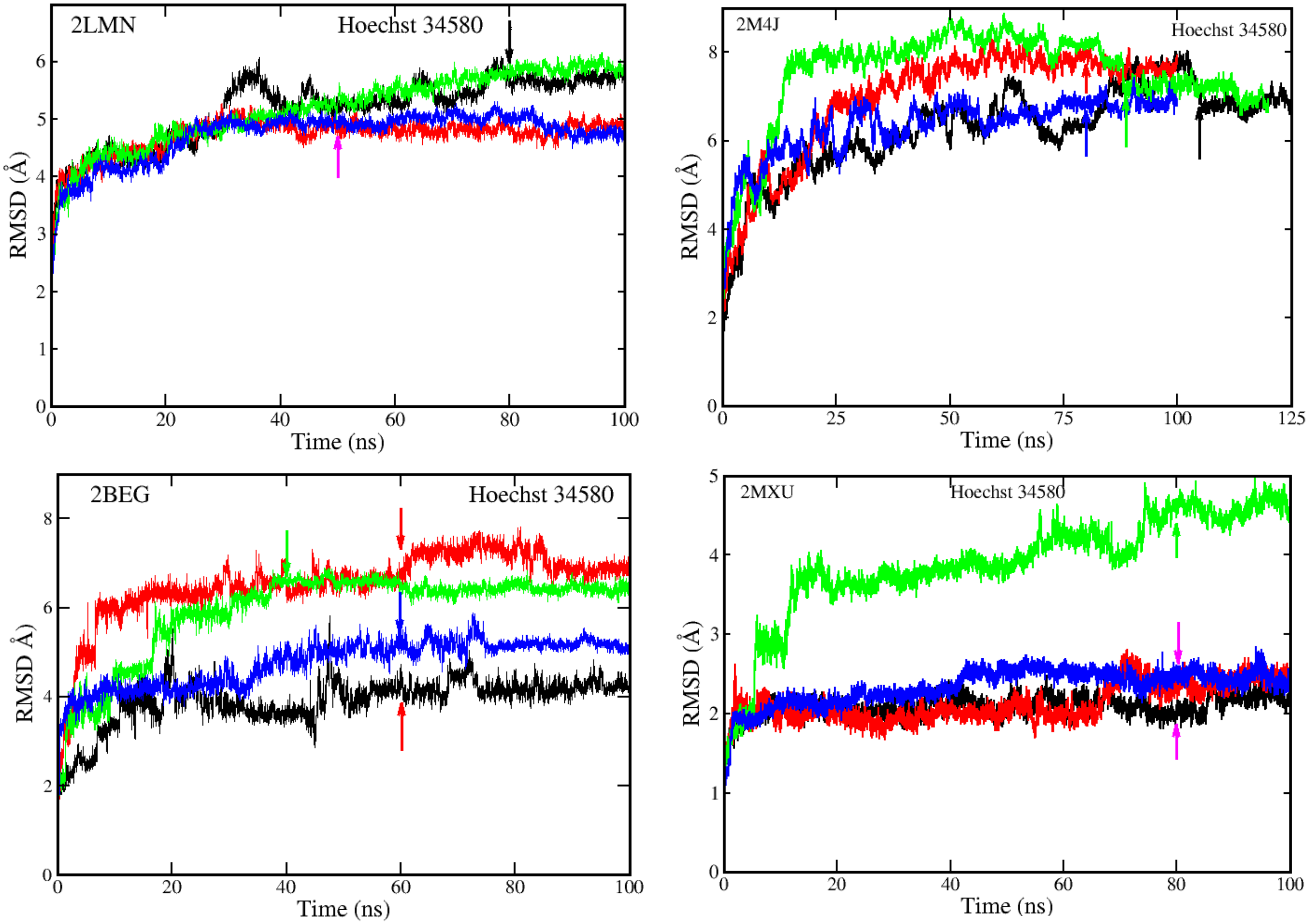

Figure S6. Time dependence of RMSD with respect to the initial structure for 2LMN, 2M4J, 2BEG and 2MXU in complex with Hoechst 34580 with the force field AMBER-f99SB-ILDN. Arrows refer to time when the systems reach equilibrium.

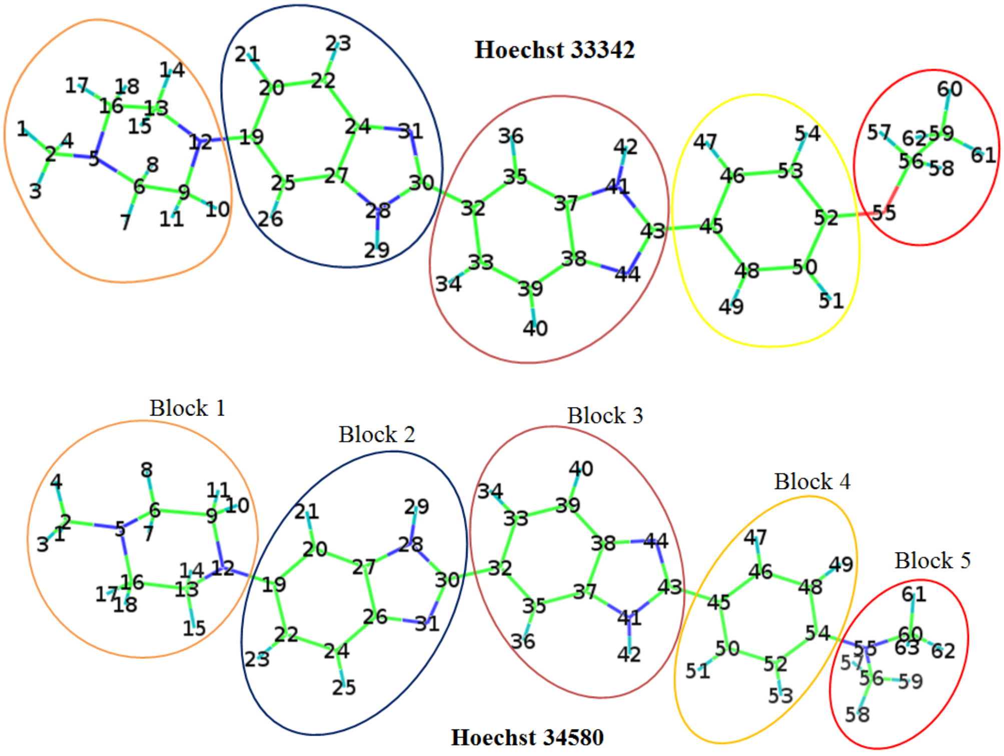

Figure S7. The structure of Hoechst 34580 and Hoechst 33342 divided into 5 blocks. Atom numbers were generated by the Gromacs software where the united atom approximation (aliphatic (CH_n) and aromatic hydrogen atoms are implicitly included by describing the C atom and attached H atoms as a single group centered on the C atom) was used.

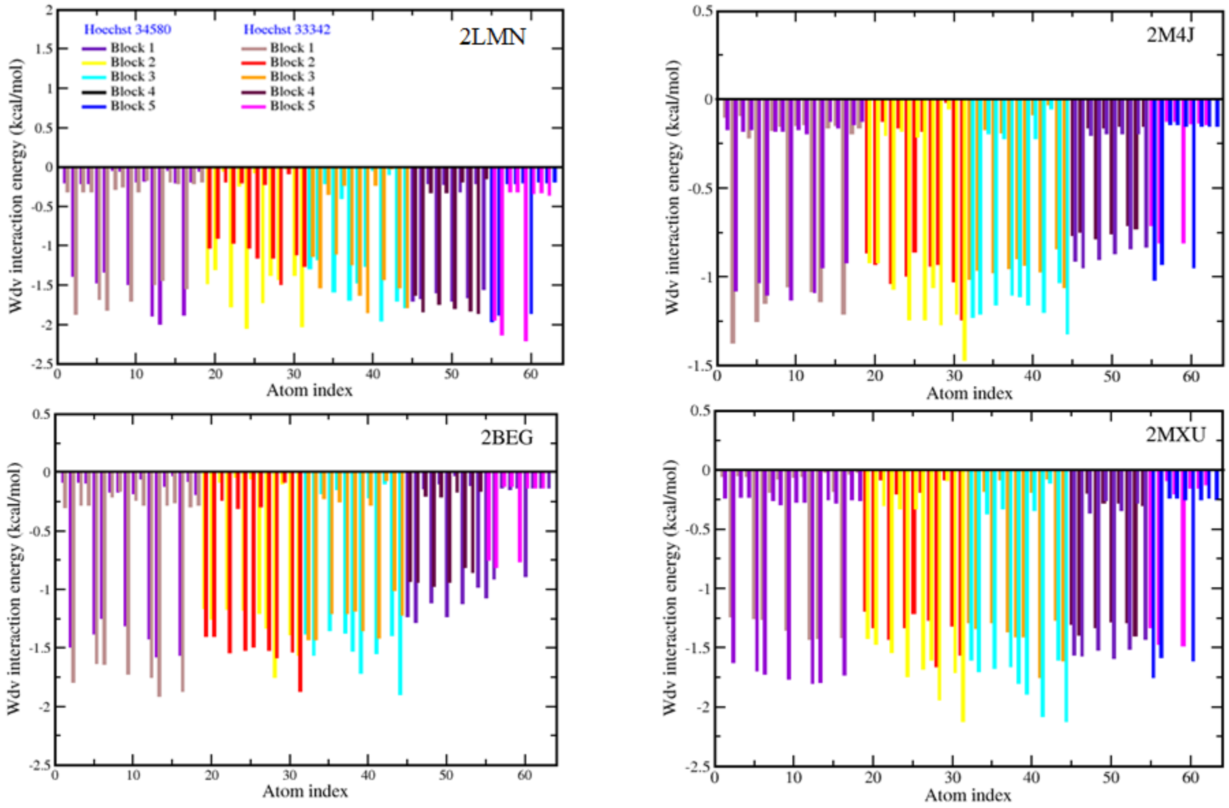

Figure S8. Per-atom contributions of DNA dyes to the vdW interaction energy with four receptors (2LMN, 2M4J, 2BEG, and 2MXU). Different colors are used to distinguish blocks of two DNA dyes.

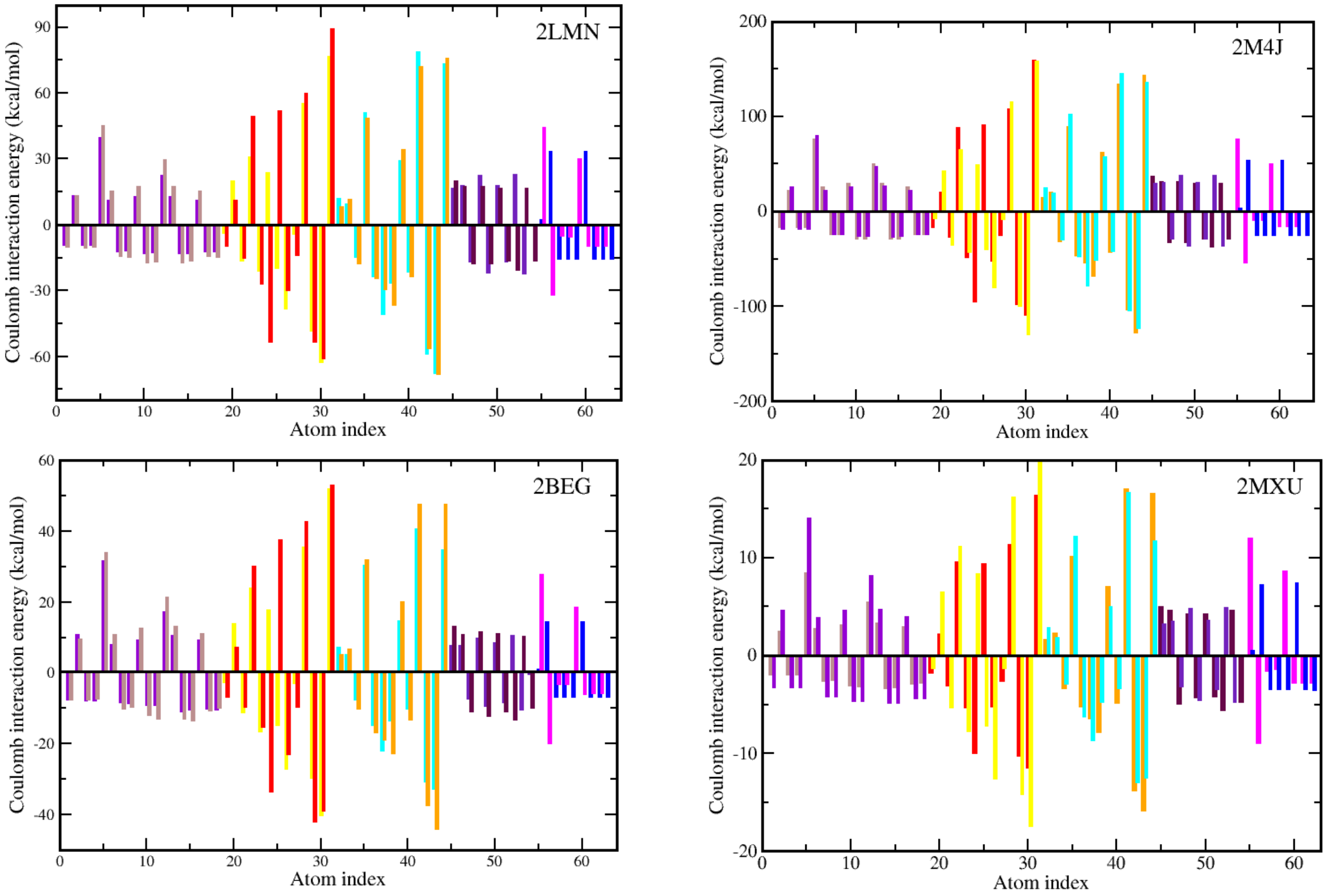

Figure S9. Per-atom contribution of ligands to the electrostatic interaction energy with four receptors (2LMN, 2M4J, 2BEG, and 2MXU). Different colors are used to distinguish blocks of two ligands (see Figure S7).

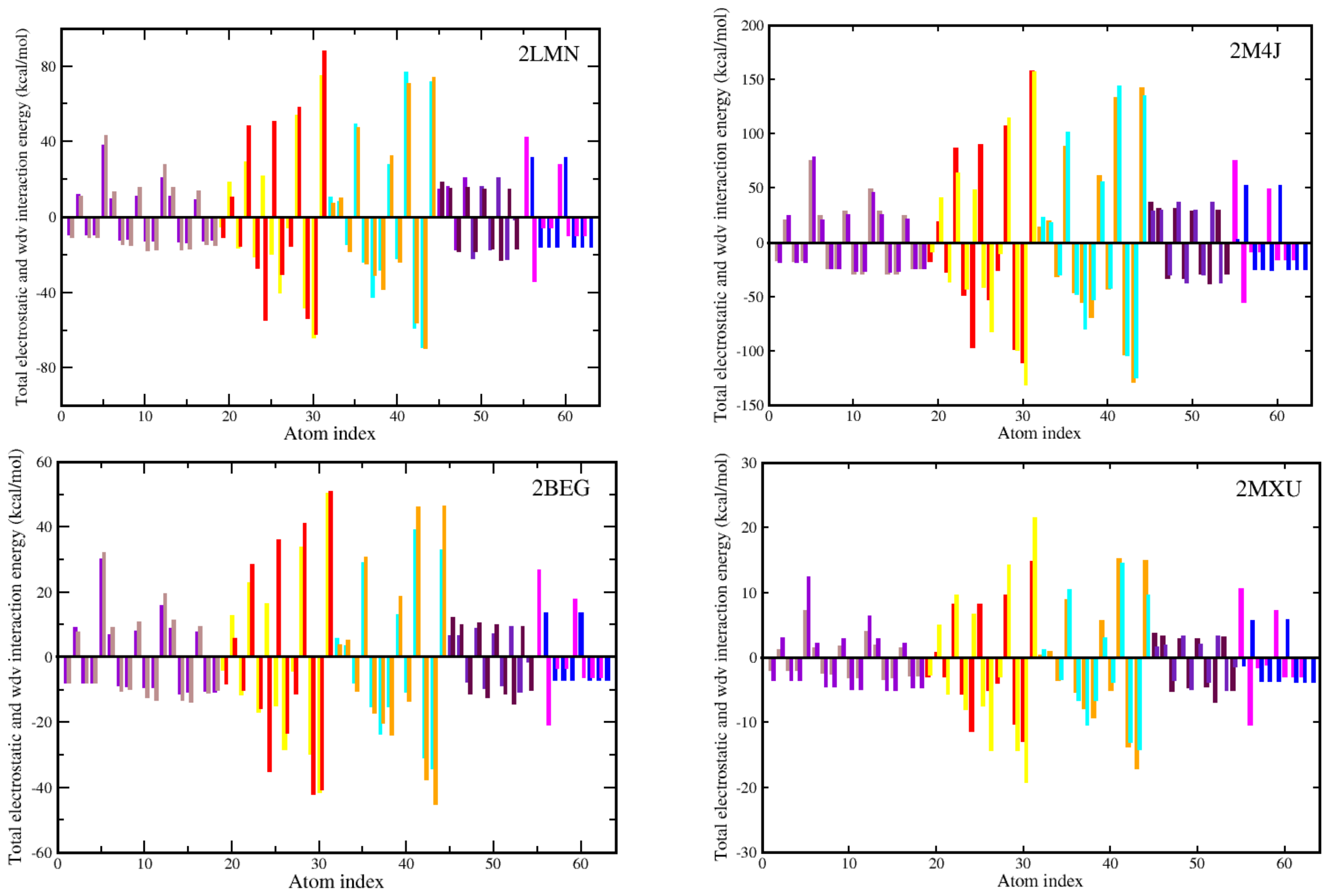

Figure S10. Per-atom total electrostatic and vdW interaction energy. Different colors are usded to distinguish blocks (see Figure S7).
